# Proteomic Analyses of Fibroblast- and Serum-Derived Exosomes Identify QSOX1 as a Marker for Non-invasive Detection of Colorectal Cancer

**DOI:** 10.3390/cancers13061351

**Published:** 2021-03-17

**Authors:** Nicole Ganig, Franziska Baenke, May-Linn Thepkaysone, Kuailu Lin, Venkatesh S. Rao, Fang Cheng Wong, Heike Polster, Martin Schneider, Dominic Helm, Mathieu Pecqueux, Adrian M. Seifert, Lena Seifert, Jürgen Weitz, Nuh N. Rahbari, Christoph Kahlert

**Affiliations:** 1 German Cancer Consortium (DKTK), German Cancer Research Center (DKFZ), D-69120 Heidelberg, Germany; Nicole.Ziegler@uniklinikum-dresden.de (N.G.); franziska.baenke@uniklinikum-dresden.de (F.B.); adrian.seifert@uniklinikum-dresden.de (A.M.S.); Lena.Seifert@uniklinikum-dresden.de (L.S.); 2 Department of Visceral, Thoracic and Vascular Surgery, University Hospital and Faculty of Medicine Carl Gustav Carus, Technische Universität Dresden, D-01307 Dresden, Germany; May-Linn.Thepkaysone@uniklinikum-dresden.de (M.-L.T.); Kuailu.Lin@uniklinikum-dresden.de (K.L.); Rao.Venkatesh@uniklinikum-dresden.de (V.S.R.); FangCheng.Wong@uniklinikum-dresden.de (F.C.W.); heike.polster@uniklinikum-dresden.de (H.P.); Mathieu.Pecqueux@uniklinikum-dresden.de (M.P.); juergen.weitz@uniklinikum-dresden.de (J.W.); 3 MS-based Protein Analysis Unit, German Cancer Research Center (DKFZ), D-69120 Heidelberg, Germany; mar.schneider@dkfz-heidelberg.de (M.S.); d.helm@dkfz-heidelberg.de (D.H.); 4 National Center for Tumor Diseases (NCT), Partner Site Dresden, D-69120 Heidelberg, Germany; 5 Department of Surgery, University Medicine Mannheim, Medical Faculty Mannheim, University of Heidelberg, Mannheim, D-68167 Mannheim, Germany; nuh.rahbari@umm.de

**Keywords:** colorectal cancer, tumour microenvironment, cancer-associated fibroblasts, extracellular vesicles, exosomes, liquid biopsy

## Abstract

**Simple Summary:**

Early diagnosis of colorectal cancer (CRC) is crucial to improve patient outcomes. The tumour microenvironment immediately adapts to malignant transformations, including the activation of fibroblasts in the connective tissue nearby. In this study, we investigated fibroblast activity-related protein secretion via extracellular vesicles (EVs). QSOX1, a protein identified to be significantly reduced in activated fibroblasts and derived EVs, was also found to be significantly reduced in circulating blood plasma EVs of CRC patients as compared to control patients. Hence, blood plasma EV-associated QSOX1 represents a promising platform for diagnostic CRC screening.

**Abstract:**

The treatment of colorectal cancer (CRC) has improved during the last decades, but methods for crucial early diagnosis are yet to be developed. The influence of the tumour microenvironment on liquid biopsies for early cancer diagnostics are gaining growing interest, especially with emphasis on exosomes (EXO), a subgroup of extracellular vesicles (EVs). In this study, we established paired cancer-associated (CAFs) and normal fibroblasts (NF) from 13 CRC patients and investigated activation status-related protein abundance in derived EXOs. Immunohistochemical staining of matched patient tissue was performed and an independent test cohort of CRC patient plasma-derived EXOs was assessed by ELISA. A total of 11 differentially abundant EV proteins were identified between NFs and CAFs. In plasma EXOs, the CAF-EXO enriched protein EDIL3 was elevated, while the NF-EXO enriched protein QSOX1 was diminished compared to whole plasma. Both markers were significantly reduced in patient-matched CRC tissue compared to healthy colon tissue. In an independent test cohort, a significantly reduced protein abundance of QSOX1 was observed in plasma EXOs from CRC patients compared to controls and diagnostic ROC curve analysis revealed an AUC of 0.904. In conclusion, EXO-associated QSOX1 is a promising novel marker for early diagnosis and non-invasive risk stratification in CRC.

## 1. Introduction

With an incidence of 6.1%, colorectal cancer (CRC) is the third most common diagnosed cancer worldwide and reflects the second leading cause of cancer-related deaths (9.2%) [1]. Numerous advancements, ranging from enhanced sensitivity of colonoscopy screenings [2] to improvements in chemotherapeutic treatment [3,4], paved the way for declining CRC incidence and mortality rates within the last decades [5,6]. Unfortunately, in approximately 21% of all diagnosed CRC cases in the US, patients present at an already advanced, metastatic stage [6]. As the 5-year survival rate for advanced CRC is reduced to only 14% compared to 90% in early localised stages [6], there is a persisting need for diagnostic strategies for early CRC diagnosis.

From the very beginning of tumour initiation, malignant cells interact with their tumour microenvironment (TME) and alter the phenotype of surrounding cells [7]. Normal fibroblasts (NFs) adjacent to the tumour transform into cancer-associated fibroblasts (CAFs) and show high abundance in the TME of CRC and other solid tumours [8]. In CRC, the amount of CAF infiltration into the tumour represented a significant prognostic marker itself [9]. Total tumour stroma and CAF-specific gene expression patterns were identified and revealed prognostic value in breast cancer [10], non-small cell lung cancer [11] and CRC [12,13]. As these signatures were examined in tissue or tissue-derived primary cells, their potential diagnostic value remains to be clarified.

During the last few years, the concept of liquid biopsy as an alternative to tissue biopsy has gained substantial interest [14]. Circulating tumour cells as well as cell-free nucleic acids have been investigated for their diagnostic and prognostic purposes in several tumour types including CRC [15,16,17,18,19]. A recent focus lies on circulating exosomes (EXO), a subtype of extracellular vesicles (EVs) that are shed from any cell type and recapitulate the topology of their cell of origin [20]. Hence, typical mutational changes that occur frequently in cancer could be detected in circulating serum EXOs of CRC patients on the mRNA level [21]. The abundance of distinct micro- and long non-coding RNAs in circulating EXOs has been shown to be altered in CRC [22,23,24], pancreatic, lung and prostate cancer patients [25,26,27] when compared to healthy or benign disease. Many studies were performed on the biological effects of EXOs on tumour cells (reviewed in [28]), revealing prominent effects of CAF-derived EXOs on tumour growth, progression and therapy response of CRC in vitro and in vivo (reviewed in [29]). Only the minority of studies investigated tumour stroma-associated biomarkers in vitro and their clinical value in vivo by analysing circulating EXOs [30,31,32], which may represent a source for diagnostic and prognostic information in gastrointestinal cancers.

The aim of this study was to investigate the potential value of fibroblast-specific markers in CRC liquid biopsies. Therefore, primary fibroblasts were isolated from CRC (referred to as CAFs) and normal tissue (referred to as NFs) of 13 CRC patients that underwent hemicolectomies. Fibroblast-derived EXO cargo was analysed by mass spectrometry, revealing 11 proteins of differential abundance in CAF-EXOs compared to NF-EXOs in initially three patients. Gene set enrichment analysis associated 7 out of the 11 proteins with epithelial-to-mesenchymal transition (EMT) and focal adhesion. Among these, NF-related QSOX1 and CAF-related EDIL3 were validated in an independent validation cohort of 10 CRC patients, in whole tissue immunohistochemistry (IHC) and in patient plasma-derived EXOs (pEXOs). QSOX1 was significantly decreased on pEXO level in CRC patients compared to vascular disease (VD) patients in an independent CRC patient cohort. Finally, diagnostic ROC curve analysis underlined the potential of NF-associated pEXO-related QSOX1 in early diagnosis of CRC.

## 2. Materials and Methods

### 2.1. Primary Fibroblast Isolation and Culture

Fresh CRC and normal colon tissues were obtained from patients undergoing hemicolectomies at the Department of Visceral, Thoracic and Vascular Surgery at the University Hospital Carl Gustav Carus Dresden between January 2017 and September 2019. Tissue collection was approved by the Ethics Committee Dresden (approval number EK76032013) and written informed consent was collected of each patient before surgery. The last follow up of all patients was carried out at the beginning of July 2020.

The tissue was rinsed intensely with 1% AntiAnti (Gibco^®^, Thermo Fisher Scientific, Waltham, MA, USA) in DPBS (Gibco^®^, Thermo Fisher Scientific) for 60 min on a rotator with frequent renewal of the washing solution. The cleansed tissue was then cut into pieces of 1 × 1 mm in size, distributed on 6-well plates and dried for 10–20 min so the tissue adhered to the plastic. The dried tissue pieces were cultured in medium composed of normal fibroblast medium (Dulbecco’s modified eagle medium (DMEM) GlutaMAX^TM^ (4.5 g/L glucose, Gibco^®^, Thermo Fisher Scientific) supplemented with 20% FBS and 1% AntiAnti) and keratinocyte serum-free medium (KSFM, Gibco^®^, Thermo Fisher Scientific) including the provided supplements, and 0,1% Primocin (InvivoGen, Toulouse, France) at a 2:1 ratio and cultured at 37 °C (310.15 K), 5% CO_2_. Within 2–4 weeks, fibroblasts grew out of the tissue pieces and when fibroblast growth was established, normal fibroblast medium was used.

### 2.2. Fibroblast Cellular Protein Expression Analysis

Equal cell numbers of each fibroblast cell line were incubated in normal fibroblast medium for 48 h and lysed with RIPA buffer (150 mM NaCl, 1% Triton X-100, 0.5% SDS, 50 mM Tris) including 1× Halt™ Protease- and Phosphatase-Inhibitor-Cocktail (Thermo Fisher Scientific). Protein concentration was determined by BCA assay (Pierce^TM^, Thermo Fisher Scientific) and equal amounts of total cellular protein were subjected to reducing (VIM, αSMA, FAPα, CAV1, CD90/Thy1, FSP1/S100A4, THBS1) or non-reducing (EDIL3, ACTN4, QSOX1) denaturation for western blot.

For the detection of proteins of interest, the following primary antibodies were used: anti-vimentin antibody [RV202] (ab8978, Abcam, Cambridge, UK), anti-Actin, α-smooth muscle antibody, mouse monoclonal (A5228, Sigma-Aldrich, St. Louis, MO, USA), human fibroblast activation protein alpha/FAP antibody (AF3715, R&D Systems, Minneapolis, MN, USA), human/mouse/rat caveolin-1 antibody (MAB5736, R&D Systems), Thy1/CD90 (D3V8A) rabbit mAb (13801, Cell Signaling Technology, Danvers, MA, USA), S100A4 (D9F9D) rabbit mAb (13018, Cell Signaling Technology), recombinant anti-quiescin Q6 antibody [EPR21866] (ab235444, Abcam), α-actinin 4 (D7U5A) rabbit mAB (15145, Cell Signaling Technology), thrombospondin-1 (D7E5F) rabbit mAb (37879, Cell Signaling Technology), human EDIL3 antibody (MAB6046, R&D Systems) and GAPDH (14C10) rabbit mAb (2118, Cell Signaling Technology). HRP-linked secondary antibodies were purchased from Cell Signaling Technology (anti-mouse IgG (7076), anti-rabbit IgG (7074)) and Bio-Rad (Hercules, CA, USA; donkey anti sheep/goat IgG (star88p)). Blots were imaged using the G Box Chemi XT4 imager (Syngene, Cambridge, UK). Graphical analyses of the western blot images were performed in ImageJ [33].

### 2.3. Exosome Isolation from Cultured Fibroblasts

For generation and isolation of EXOs, fibroblasts were expanded in 875 m^2^ multiflasks (Falcon^®^, Corning^®^, Corning, NY, USA). When the cells reached a density of 90–95%, the flasks were washed with DPBS (Gibco^®^, Thermo Fisher Scientific) prior to addition of 100 mL starvation medium (DMEM GlutaMAX^TM^ supplemented with 0.4% BSA, Sigma-Aldrich). After 48 h, the conditioned medium was collected by decanting and subjected to differential centrifugation combined with ultrafiltration. First, the conditioned medium was spun at 2000× *g* and 4 °C (277.15 K) for 30 min, followed by filtration using a 0.2 µm PES filter. Next, VivaSpin columns with a 100,000 MWCO PES exclusion membrane were used to concentrate the EXOs upon conditioned medium centrifugation at 3000× *g.* Finally, the EXO concentrate was diluted with PBS to a final volume of 35 mL and subjected to overnight ultracentrifugation at 100,000× *g* and 4 °C (277.15 K). Depending on the downstream analysis, the EXO pellet was processed differently. For naïve EXO isolation, the supernatant was discarded carefully, leaving 4 mL at the bottom to resuspend the EXOs. For TEM analysis or protein isolation, the supernatant was discarded completely and the EXO pellet was solved in PBS or lysed in RIPA buffer including 1× Halt™ Protease- and Phosphatase-Inhibitor-Cocktail. The generated samples were stored on ice until further processing.

### 2.4. Characterisation of Exosomes

Characterisation of the isolated EXOs was performed using nanoparticle tracking analysis (NTA) and transmission electron microscopy (TEM). NTA analysis of particle size and concentration was performed using ZetaView^®^ (Particle Metrix, Inning am Ammersee, Germany) immediately after isolation. For TEM imaging, EXOs at an optimal concentration were absorbed onto 400-mesh carbon/formvar grids, rinsed with PBS and ddH_2_O, air dried and counterstained using uranyl acetate. Images were taken at a Tecnai Bio Twin transmission electron microscope (Field Electron and Ion Company (FEI), Hillsboro, OR, USA) equipped with an AMT CCD Camera (Advanced Microscopy Techniques, Woburn, MA, USA).

In addition, immunoblot analyses were performed on EXOs lysed in RIPA buffer. Protein concentration was determined by BCA assay (Pierce^TM^, Thermo Fisher Scientific) and equal amounts of total vesicular protein were subjected to non-reducing denaturation for western blot. For the detection of proteins of interest, the following primary antibodies were used: recombinant anti-CD9 antibody [EPR2949] (ab92726, Abcam, 1:200), anti-CD63 antibody (ab68418, Abcam, 1:500), CD81 antibody (NBP2-20564, Novus Biologicals, Centennial, CO, USA, 1:400), anti-TSG101 antibody [4A10] (ab83, Abcam, 1:500), Flotillin-1 antibody (3253, Cell Signaling Technology, 1:500), calreticulin antibody (2891, Cell Signaling Technology, 1:1000), recombinant anti-Quiescin Q6 antibody [EPR21866] (ab235444, Abcam, 1:1000), α-actinin 4 (D7U5A) rabbit mAB (15145, Cell Signaling Technology, 1:1000), thrombospondin-1 (D7E5F) rabbit mAb (37879, Cell Signaling Technology, 1:200) and human EDIL3 antibody (MAB6046, R&D Systems, 1:500). HRP-linked secondary antibodies were purchased from Cell Signaling Technology (anti-mouse IgG (7076), anti-rabbit IgG (7074)). Blots were imaged at a G:Box Chemi XT4 imager (Syngene).

### 2.5. Mass Spectrometric Analysis of EXO Cargo

#### 2.5.1. Sample Preparation

Vesicular protein lysates were loaded on a SDS-PAGE-gel and ran 0.5 cm in the gel. After coomassie staining the sample underwent tryptic digestion according to the protocol by Shevchenko et al. [34] adapted to a DigestPro MSi robotic system (INTAVIS Bioanalytical Instruments AG, Köln, Germany).

#### 2.5.2. Mass Spectrometry

Dried peptides were resuspended in reconstitution buffer (2.5% 1,1,1,3,3,3-hexafluoro-2-propanol, 0.1 % TFA in water) prior to LC-MS measurement, which was conducted using a Ultimate 3000 UPLC (Thermo Fisher Scientific) coupled to an Q-Exactive HF-X mass spectrometer (Thermo Fisher Scientific). During the LC separation the peptides were first loaded onto a trapping cartridge (Acclaim PepMap300 C18, 5 µm, 300 Å wide pore, Thermo Fisher Scientific, and washed for 3 min with 0.1% TFA in water. Analytical separation was performed using a nanoEase MZ Peptide analytical column (300 Å, 1.7 µm, 75 µm × 200 mm, Waters, Milford, MA, USA) and carried out for either 210 min or 90 min total analysis time. The 210 min method consisted of a three-step gradient going from 2–8% solvent B (80% acetonitrile, 20% water with 0.1% formic acid) in 15 min, 8–25% in 135 min and 25–40% in 30 min followed by a washing and an equilibration step. Solvent A being water and 0.1 % formic acid. The 90 min method included an elution step ramping solvent B content from 5–35% in 72 min followed by a washing and equilibration step. Eluting peptides were analyzed online by a coupled Q-Exactive-HF-X mass spectrometer running in data-depend acquisition mode. MS settings are described for the 210 min method and in brackets are the variations for the 90 min method. Full scans were performed at 120 k (60 k) resolution on a mass range covering 375–1500 m/z with a maxIT of 54 ms (45 ms). Followed by up to 35 MSMS scans (1 s cycle time) at 15 k resolution with a maxIT of 22 ms (54 ms) for up to 1 × 10^5^ ions AGC target. Precursors were isolated with a window of 1.6 m/z and fragmented collision energy of 27 NCE). Unassigned and singly charged peptides have been excluded from fragmentation and dynamic exclusion was set to 60 s (30 s).

#### 2.5.3. Data Analysis

Data analysis was carried out by MaxQuant (version 1.6.14.0) [35] using an organism specific database extracted from Uniprot [36] under default settings. Identification FDR cutoffs were 0.01 on peptide level and 0.01 on protein level. Match between runs option was enabled to transfer peptide identifications across Raw files based on accurate retention time and m/z. Quantification was done using a label free quantification approach based on the MaxLFQ algorithm [37]. A minimum of two quantified peptides per protein was required for protein quantification.

#### 2.5.4. Statistics and Further Analyses

Data have been further processed by in-house compiled R-scripts to plot and filter data and the Perseus software package (version 1.6.7.0, Max-Planck-Institute of Biochemistry, Martinsried, Germany) using default settings for further imputation of missing values and statistical analysis [38]. Application of paired t test identified proteins of differential abundance in NF- vs. CAF-EXOs (*q* < 0.05, difference > |1.0|). Identified proteins with more than four undefined values in total or more than three undefined values in the NF/CAF subgroups were excluded. To identify proteins of interests, gene set enrichment analysis [39,40] and TCGA CRC dataset examination [41] was performed.

### 2.6. Immunohistochemistry

CRC and normal colon tissue were obtained by surgery, processed and embedded in paraffin at the pathology department of the University Hospital Dresden. 8 µm thick slides were cut using a microtome and placed on glass slides (Superfrost^®^ Plus, Thermo Scientific). Following dewaxing in xylene and rehydration in a decreasing ethanol gradient (100%, 96%, 85%, 70%), slides were briefly washed in 0.1% Tween 20 in Tris-buffered saline (TBS-T, pH 7.6) and endogenous peroxidase was quenched using 3% H_2_O_2_/TBS-T. After washing, slides were incubated in 0.01 M sodium citrate buffer supplemented with 0.05 % Tween 20 (pH 6.0) upon alternating calefaction in a microwave to ensure antigen retrieval, followed by another washing step. Next, slides were blocked with 5 % goat serum (Sigma-Aldrich)/1% BSA/TBS-T for 60 min in a humidified chamber at room temperature. Excess liquid was discarded and without washing, primary antibody diluted in 1% BSA/TBS-T was added and incubated overnight at 4 °C (277.15 K). The following primary antibodies were used: anti-Actin, α-smooth muscle antibody, mouse monoclonal (A5228, Sigma-Aldrich, 1:1000), human EDIL3 antibody (MAB6046, R&D Systems, 1:200), recombinant anti-quiescin Q6 antibody [EPR21866] (ab235444, Abcam, 1:200), mouse (E5Y6Q) mAb IgG2a (61656, Cell Signaling Technology, 1:200) and rabbit (DA1E) mAb IgG XP^®^ isotype control (3900, Cell Signaling Technology, 1:200). Next, slides were treated with SignalStain^®^ Boost IHC detection reagent (HRP, rabbit (8114) or mouse (8125), Cell Signaling Technology) for 30 min, washed, and treated with ImmPACT^®^ DAB substrate (SK-4105, Vector Laboratories, Burlingame, CA, USA). Within 1–10 min, an appropriate staining was achieved, and slides were briefly washed in tap water, followed by Hematoxylin/Mayer counterstaining and the administration of an increasing ethanol gradient (70%, 85%, 96%, 100%) and xylene. Finally, the slides were mounted in Entellan (1079600500, Sigma-Aldrich), covered with cover slips (Engelbrecht, Edermünde, Germany) and air dried. H&E staining was performed at the pathology department of the University Hospital Dresden.

Slides were digitalised using a Panoramic SCAN (3D Histech, Budapest, Hungary) and viewed in CaseViewer (3D Histech, software version 2.4). Of each slide, three representative pictures were exported and loaded into QuPath (software version 0.2.1) [42]. A minimum of three stained and three unstained areas were annotated manually to create target-specific pixel classifiers (Figure S1). During automated batch processing, positively stained areas for each image were annotated and the particular area was measured.

### 2.7. Protein Isolation of Plasma-Derived EXOs

Blood samples were isolated from patients undergoing surgical intervention at the Department of Visceral, Thoracic and Vascular Surgery at the University Hospital Carl Gustav Carus Dresden immediately before surgery (approved by the Ethics Committee Dresden, approval number EK76032013). Venous blood samples were collected in EDTA containing monovettes and processed within 60 min. The blood was transferred to a 15 mL falcon tube and centrifuged twice at 1500× *g* for 12 min followed by supernatant collection. The retrieved plasma was distributed to cryotubes (1 mL aliquots) and stored at −80 °C (193.15 K).

For EXO isolation, 600 µL plasma were thawed on ice and cleared by sequential centrifugation for 20 min at 2000× *g* and 10,000× *g*, transferring the supernatant into a fresh tube after each step. 50 µL of clarified plasma were directly mixed with RIPA buffer to isolate whole plasma protein (wP). 250 µL DPBS (Gibco^®^, Thermo Fisher Scientific) was added to the remaining 500 µL of clarified plasma and mixed by vortexing, followed by adding 150 µL Exosome Precipitation Reagent (Total Exosome Isolation (from plasma), Invitrogen, Thermo Fisher Scientific). Samples were vortexed again, incubated at room temperature for 10 min, and centrifuged for 5 min and another 30 sec at 10,000× *g*, collecting the supernatant after each centrifugation step (exosome-depleted plasma (edP)). Five hundred µL of DPBS was added to the remaining EV pellet, and the samples were placed on a horizontal shaker at 1400 rpm for 15–20 min for the pellet to resolve. Finally, Vivaspin500 columns with a 100,000 MWCO cutoff PES membrane (Invitrogen, Thermo Fisher Scientific) were applied to reduce the sample volume to 100 µL or less by ultrafiltration at 13,000× *g* for at least 30 min. Sample volume was adjusted to 100 µL by addition of DPBS if necessary. 50 µL of the plasma-EXO solution were subjected to protein isolation using RIPA buffer, and the remaining sample was subjected to NTA using ZetaView^®^ (Particle Metrix) to evaluate particle size and concentration.

### 2.8. ELISA

Human QSOX1 (sulfhydryl oxidase 1) ELISA Kit (E-EL-H5503, Elabscience, Houston, TX, USA) was used to investigate plasmatic vesicular protein abundance of QSOX1 in a test cohort of CRC patients and controls, according to the manufacturer’s instructions. Total target protein content in 1 × 10^9^ EXOs was calculated based on the individual NTA measurements, total protein concentrations and ELISA results.

### 2.9. Statistical Analysis

Statistical analyses were performed in GraphPad Prism (GraphPad Software, San Diego, CA, USA), unless otherwise noted. Mann-Whitney-U test was applied to investigate differences in the protein abundances evaluated by western blot, IHC and ELISA, and diagrams show median with interquartile range. Diagnostic ROC curves and Youden Index were computed to clarify the diagnostic value of pEXO-associated QSOX1 in CRC. *p* values ≤ 0.05 were considered statistically significant.

## 3. Results

### 3.1. Fibroblast Activation Status Can Be Detected at the Cellular and Vesicular Protein Level

**Figure 1:**
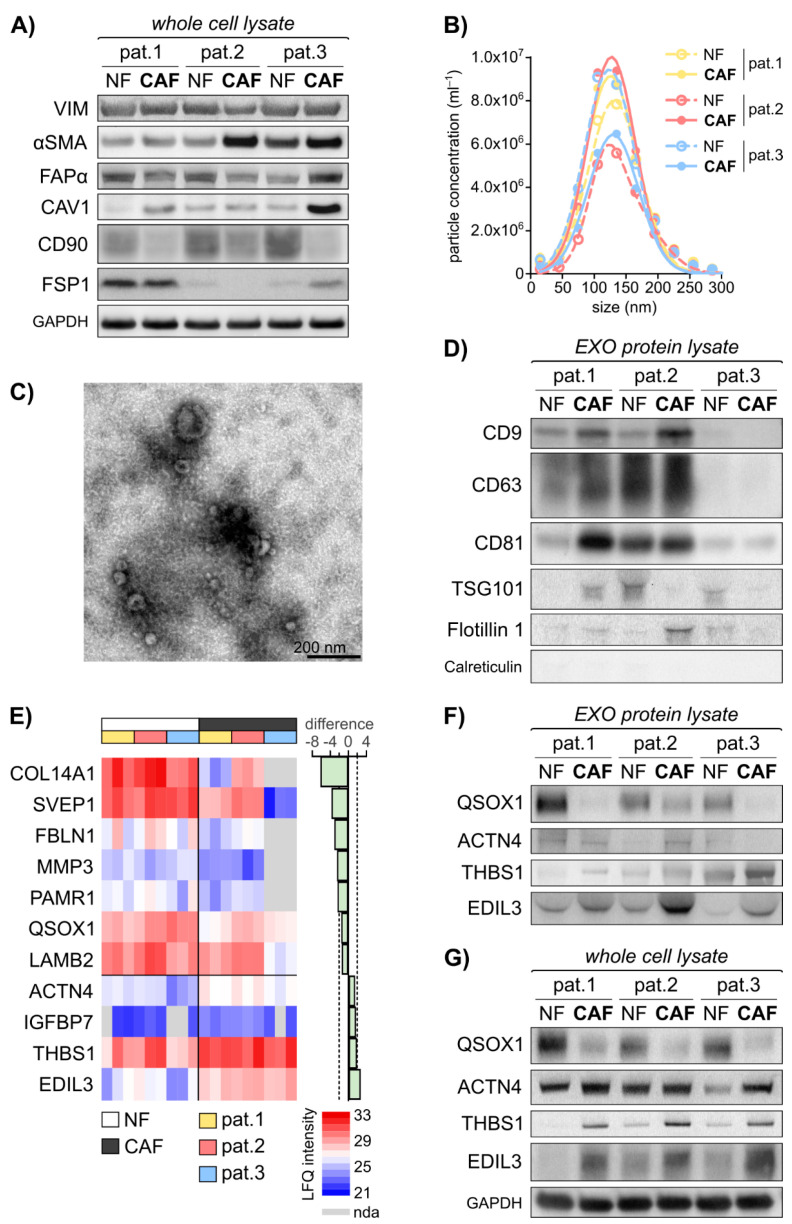
Primary fibroblast-derived EXOs display an activation status dependent protein cargo. The established fibroblast cell lines were subjected to cellular protein isolation after 48 h incubation of equal cell numbers in normal growth medium. EXOs were isolated using combined differential centrifugation and ultrafiltration approach following 48 h of fibroblast incubation in starvation medium. Exosomal protein was isolated in biological triplicates and subjected to Mass Spectrometry. Statistical Analysis was performed in Perseus. (**A**) Immunoblot analysis of vimentin (VIM), α-smooth-muscle actin (αSMA), fibroblast activation protein α (FAPα), caveolin 1 (CAV1), cluster of differentiation 90 (CD90)/Thy1 and fibroblast-specific protein 1 (FSP1) in primary fibroblasts, including GAPDH as loading control. (**B**) Particle size distribution in the isolated EXOs (mean of *n* = 4–10) as measured by nanoparticle tracking analysis (NTA) using ZetaView^®^. (**C**) Representative transmission electron microscopy (TEM) image of fibroblast-derived EXOs. (**D**) Immunoblot analysis of EXO markers CD9, CD63, CD81, Flotillin 1 and Tumor susceptibility 101 (TSG101), including calreticulin as negative control. (**E**) Heat map of mass spectrometry data illustrating significantly deregulated vesicular proteins. Proteins with more than 4 undefined values in total or more than 3 undefined values in the NF/CAF subgroups are excluded. Paired *t* test: *q* < 0.05, diff. > |1.0|. LFQ: label-free quantification; nda: no data acquired. (**F**) Immunoblot analysis of quiescin sulfhydryl oxidase 1 (QSOX1), actinin α4 (ACTN4), thrombospondin 1 (THBS1) and EGF-like repeats and discoidin domains 3 (EDIL3) in EXOs. (**G**) Immunoblot analysis of QSOX1, ACTN4, THBS1 and EDIL3 in primary fibroblasts whole cell lysate, including GAPDH as loading control. The images of uncropped western blot figures are shown in Figure S8.

To investigate alterations in fibroblasts of the TME upon interaction with CRC cells, CAFs were successfully established from malignant colorectal tissue of three CRC patients following surgical resection (Table S1 and Figure S2). Patient-matched quiescent NFs were isolated from healthy colon tissue of the hemicolectomy specimens. Protein expression analysis was performed on this discovery cohort to characterise the primary cell lines with emphasis on the fibroblast activation status (Figure 1A). The expression of vimentin (VIM) was detected in all six established cell lines, and an increased expression of α smooth muscle actin (αSMA) in a subset of patient-derived CAFs compared to the respective patient-matched NFs, illustrating their enhanced activity status. Of note, induced levels of fibroblast activation protein α (FAPα) in CAFs were only detected in patient 3. Instead, caveolin-1 (CAV1) showed strong abundance in CAFs, while cluster of differentiation 90 (CD90)/Thy1 was increased in NFs. Fibroblast specific protein 1 (FSP1) could only be detected in three established fibroblast lines, showing heterogeneity in the fibroblast cell lines that is independent of their activation status. Taken together, the established cell lines comprise fibroblasts, and CAFs derived from the cancer site show an induced activation status compared to the respective patient-matched NFs.

EXOs are shed by every cell of the human body and their load reflects the cell of origin. Therefore, we hypothesised that fibroblast activity markers could be found in fibroblast-derived EXOs. EXOs were isolated from the six patient-derived fibroblast cell lines by combined ultrafiltration and differential centrifugation and subjected to further analysis. The particles isolated from the fibroblast conditioned cell culture medium exhibited an overall median size of 138.9 nm (114.9–157.9 nm) with an overall median concentration of 4.5 × 10^9^ particles/mL (3.3 × 10^8^–1.1 × 10^10^ particles/mL) in nanoparticle tracking analysis (NTA) (Figure 1B). In transmission electron microscopy (TEM), the particles showed the typical cup shape with a size range of 50–150 nm (Figure 1C). Additionally, at least 3 of the exosomal markers CD9, CD63, Flotillin 1 and Tumor susceptibility 101 (TSG101) were identified in the isolated particles, with varying expression, depending on their cell line of origin (Figure 1D). Interestingly, EXO biogenesis and secretion may itself be altered during fibroblast activation, as these markers show differential abundance in patient matched NFs and CAFs. The expression of common EXO proteins in patient 3 was lower compared to patient 1 and 2. The luminal ER marker calreticulin was absent in all samples, illustrating the clearance of cellular content during EXO isolation. In conclusion, we were able to identify fibroblast activation specific protein patterns and successfully isolated EXOs derived from CAFs and matched NFs.

### 3.2. EXO-Related Fibroblast Activity Patterns Revealed by Mass Spectrometry

To examine differences in the protein content of NF- and CAF-derived EXOs, we performed mass spectrometry of these primary fibroblast-derived EXOs in biological triplicates. A total of 825 proteins were identified in the analysed samples (Figure S3, Table S2). Proteins with more than four undefined values in total or more than three undefined values in the NF/CAF subgroups were excluded. Statistical analysis using paired t test identified 11 proteins of significantly different abundance (*q* < 0.05, difference > |1.0|) in CAF- vs. NF-derived EXOs. Figure 1E displays the label free quantitative (LFQ) intensities of these 11 proteins in the NF- and CAF-EXOs samples as identified by mass spectrometry. To get insight into the impact of the activation status-related protein patterns identified by mass spectrometry analysis, gene set enrichment analysis was performed of the 11 proteins and the results are summarised in Table S3. Five proteins were associated with the hallmark gene set of epithelial-to-mesenchymal transition (EMT) and three proteins were implicated in the KEGG pathway of focal adhesion.

Among these EMT and focal adhesion related proteins, fibulin 1 (FBLN1), EGF-like repeats and discoidin domains 3 (EDIL3), matrix metallopeptidase 3 (MMP3) and thrombospondin 1 (THBS1) show the strongest fold change (absolute difference of 3.16, 2.71, 2.51 and 1.91) (Figure 1E). The most significant deregulation is shown by EDIL3, MMP3, actinin α4 (ACTN4) and quiescin sulfhydryl oxidase 1 (QSOX1) (*q* values: 0.000; 0.001; 0.002; 0.002) (Figure 1E). Except for MMP3, these proteins were detected in at least 50 independent experiments in human EXOs, as listed in Vesiclepedia [43,44] and display a significantly decreased mRNA expression in CRC compared to healthy colorectal tissue in the TCGA CRC mRNA expression dataset (Figure S4) using Oncomine [41]. All six proteins are differentially expressed in malignant vs. healthy tissue in CRC (FBLN1 [45,46], MMP3 [47,48,49], ACTN4 [50,51], THBS1 [52], EDIL3 [53]) or other tumour entities (QSOX1 [54,55]). The NF-EXO enriched protein QSOX1 and the CAF-EXO enriched proteins ACTN4, THBS1 and EDIL3 were taken forward and subjected to validation. At first, an immunoblot analysis of the four chosen candidate biomarkers in both exosomal and cellular protein lysates of the six established fibroblast cell lines was conducted. On the exosomal level, the immunoblot validated the decrease of QSOX1 as well as the increase of THBS1 and EDIL3 in CAF-EXOs compared to matched NF-EXOs in patients 1–3 (Figure 1F). The expression on the cellular protein level correlated with the exosomal expression as QSOX1 showed increased levels while THBS1 and EDIL3 were decreased in NFs (Figure 1G). However, ACTN4 levels were not confirmed to be higher expressed in CAF-EXOs (Figure 1F).

To summarise, we were able to identify activation status-related proteins in fibroblast-derived EXOs by using mass spectrometry and validated three of the four selected candidate proteins (QSOX1, THBS1 and EDIL3) related to malignant transformation and tumour progression.

### 3.3. Fibroblast-Derived Vesicular Proteins in CRC Solid and Liquid Biopsies

As we aimed to identify potential biomarkers for diagnostic purposes in vitro and to assess these markers in vivo, we investigated the QSOX1, THBS1 and EDIL3 levels in blood plasma obtained from patients 1–3 prior to tumour resection. To explore the expression of the investigated markers, we analysed the plasma (whole plasma, wP), plasma-derived EXOs (pEXO) and the supernatant remaining after isolating the EXOs from the plasma, referred to as EXO-depleted plasma (edP).

**Figure 2:**
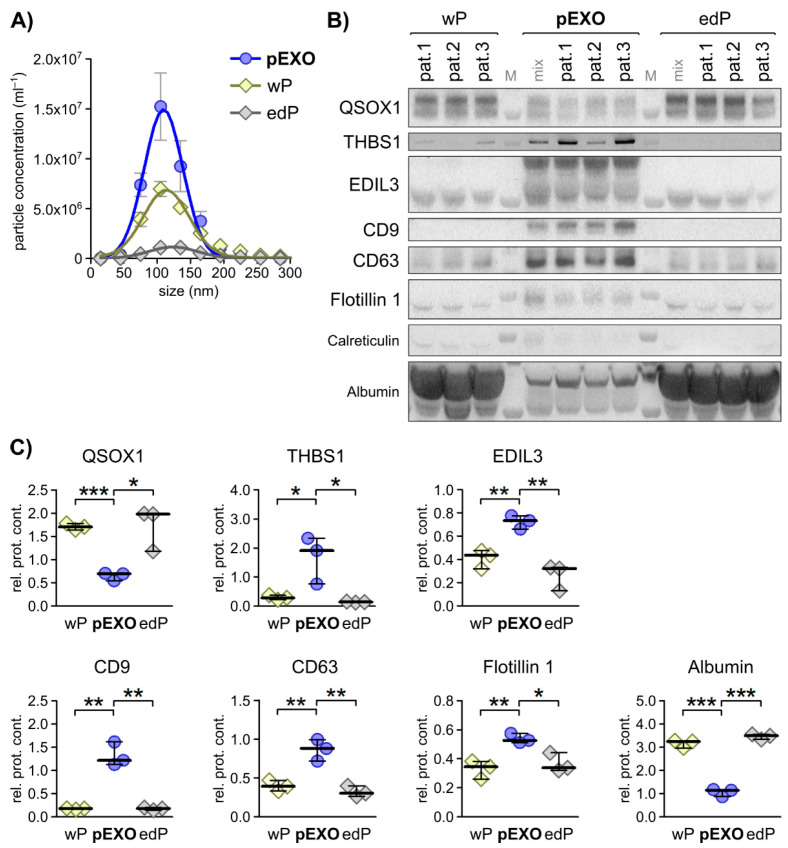
Selected primary fibroblast activation status dependent EXO markers display specificity to blood EXOs in matched CRC patient plasma. (**A**) Particle size distribution of patient-matched plasma EXOs (pEXO) in comparison to whole plasma (wP) and EXO-depleted plasma (edP) as measured by NTA. (**B**) Immunoblot analysis of quiescin sulfhydryl oxidase 1 (QSOX1), thrombospondin 1 (THBS1), EGF-like repeats and discoidin domains 3 (EDIL3), the EXO markers CD9, CD63 and Flotillin 1, including calreticulin as negative control, and albumin in wP, pEXO and edP protein lysates. (**C**) Graphical analysis of immunoblots shown in (B) using ImageJ, comparing signal strength to pEXO mix. Unpaired *t*-test: * *p* < 0.05, ** *p* < 0.01, *** *p* < 0.001.

**Figure 3:**
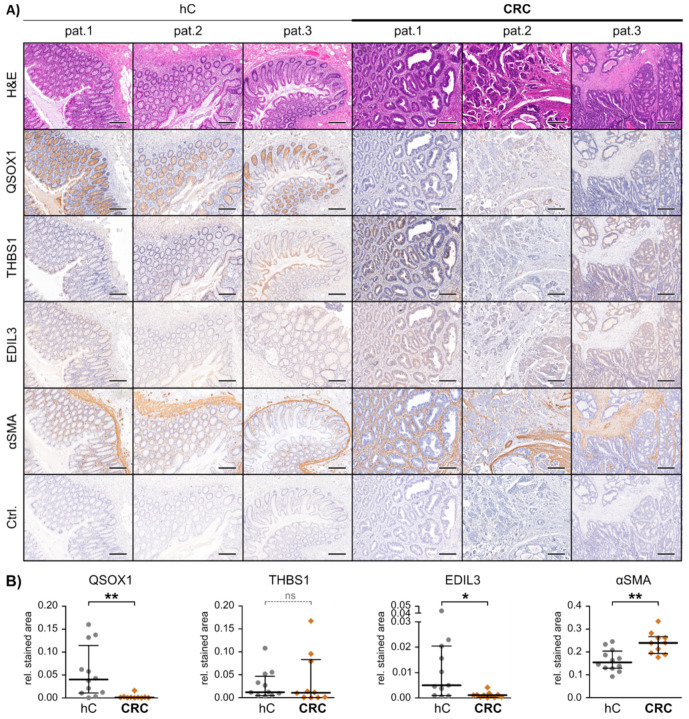
In vivo marker expression in patient-matched healthy and malignant colon tissue. (**A**) Representative images of paraffin embedded tissue slides of healthy (hC) and malignant (CRC) colon tissue derived from patients 1–3, hematoxylin and eosin (H&E) or immunohistochemically stained for the proteins quiescin sulfhydryl oxidase 1 (QSOX1), thrombospondin 1 (THBS1), EGF-like repeats and discoidin domains 3 (EDIL3), α-smooth-muscle actin (αSMA) and IgG control (Ctrl.). Scale bars equal 250 µm. (**B**) Graphical IHC staining analysis performed in QuPath. From each patient and tissue, a minimum of three representative areas were subjected to graphical and statistical analysis. Mann-Whitney-U test: * *p* < 0.05, ** *p* < 0.01. ns = not significant.

NTA analysis revealed significant differences in the EXO concentrations of these three fractions, with pEXO showing the highest and edP showing the lowest particle concentration (Figure 2A), with a consistent size distribution (50–150 nm) throughout all fractions. Immunoblot analysis of these fractions was performed and the EXO markers CD9, CD63 and Flotillin-1 demonstrated highest abundance in the pEXO fraction, while albumin was markedly decreased in pEXO compared to wP and edP (Figure 2B,C). These results suggest successful enrichment of EXOs from patient plasma with low contamination. In addition, the candidate CAF-specific proteins THBS1 and EDIL3 demonstrated significantly higher abundance in the pEXO fraction (Figure 2B,C), whereas the candidate NF-specific protein QSOX1 showed significantly reduced abundance in pEXOs. Since blood-derived EXOs display a heterogeneous population originating from any cell of the human body, we determined the expression of the three proteins of interest in tissue sections of normal and cancerous colorectal tissue of patients 1–3 using immunohistochemical staining (IHC). We were able to detect all markers in normal and malignant colorectal tissue with inter-patient heterogeneity in intensity (Figure 3A). A graphical staining analysis was performed in QuPath^®^, highlighting a significant reduced staining intensity in CRC tissue for QSOX1 and EDIL3 (Figure 3B) in accordance with the mRNA expression data of the TCGA CRC cohort (Figure S4).

In summary, the pEXO protein content correlates to our findings of fibroblast-derived EXO levels of QSOX1, THBS1 and EDIL3, and IHC revealed significantly different expression of QSOX1 and EDIL3 in malignant compared to healthy colon tissue. This demonstrates the potential of the three markers to estimate tumour burden in blood samples of CRC patients.

### 3.4. Identification of EXO-Bound QSOX1 as a Non-Invasive Biomarker for Diagnosis and Risk Stratification in Colorectal Cancer

To further validate the impact of the three candidate biomarkers QSOX1, THBS1 and EDIL3 in CRC, a validation cohort of matched NFs and CAFs was established from additional 10 CRC patients (pat. 4–13) (Table S1 and Figure S2).

First, protein expression analysis was performed in this validation cohort to characterise the activation status of the primary fibroblasts (Figure S5). Upon comparison of all CAFs with all NFs, the two proteins αSMA and CAV1 showed a clear trend towards increased abundance in CAFs, while CD90 was significantly enriched in NFs. The expression of FAPα showed heterogeneity throughout the primary cell lines, and FSP1 could only be detected in three additional fibroblast cell lines. Taken together, we observed a heterogeneity in the expression of fibroblast markers in our cohort and three proteins (αSMA, CAV1 and CD90) are suitable markers for determining fibroblast activation status to differentiate CAFs from NFs in colorectal cancer.

**Figure 4:**
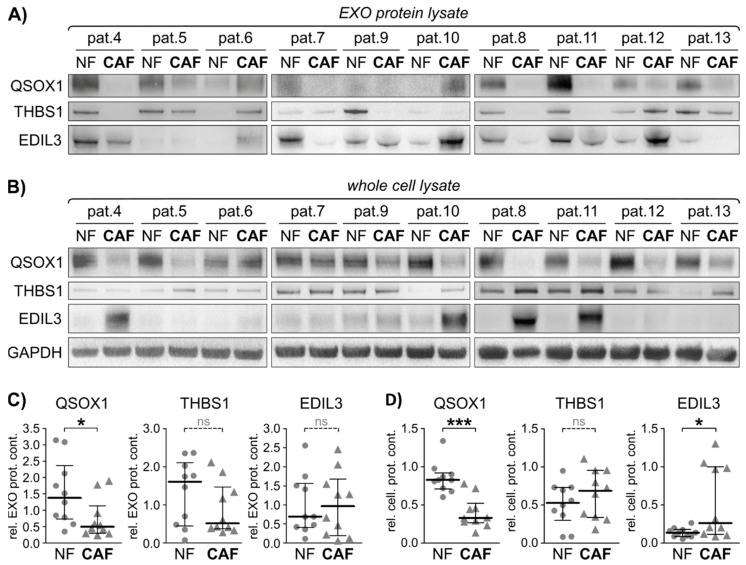
Exosomal fibroblast activity marker validation in an independent validation cohort. Twenty additional fibroblast cell lines derived from 10 CRC patients were subjected to cellular protein and EXO isolation. Exosomal protein was isolated and subjected to immunoblot. (**A**) Immunoblot analysis of quiescin sulfhydryl oxidase 1 (QSOX1), thrombospondin 1 (THBS1), EGF-like repeats and discoidin domains 3 (EDIL3), in primary fibroblasts-derived EXOs. (**B**) Immunoblot analysis of QSOX1, THBS1 and EDIL3 in primary fibroblasts whole cell lysate, including GAPDH as loading control. (**C**) Graphical analysis of EXO protein immunoblots shown in (**A**) using ImageJ, relative to GAPDH. Mann-Whitney-U test: * *p* < 0.05. (**D**) Graphical analysis of cellular protein immunoblots shown in (**B**) using ImageJ, relative to GAPDH. Mann-Whitney-U test: * *p* < 0.05, *** *p* < 0.001. ns = not significant.

Second, EXOs were isolated from these independent primary fibroblast cell lines (10 NF and 10 CAF) and protein lysates were subjected to immunoblot analysis for the three proteins of interest identified. The vesicular protein content of QSOX1 was significantly reduced in CAF-EXOs compared to NF-EXOs (Figure 4A,C), which was consistent on the cellular level (Figure 4B,D). EDIL3 reached significance in whole cell lysate but not in the EXO protein fractions, while THBS1 did not show a significant difference in NF vs. CAF in both analyses. The expression of the three proteins was also evaluated in whole tissue slides using IHC and significantly decreased staining intensities of QSOX1 and EDIL3 were observed in malignant tissue compared to patient matched normal tissue (Figure S6), and THBS1 again did not display differential protein expression between colon and CRC similar to patients 1–3.

In conclusion, QSOX1 and EDIL3 were significantly different between NFs and CAFs in patients with CRC. In particular, QSOX1 was the most robust EXO-related protein in this study and could provide a pivotal biomarker in CRC.

**Figure 5:**
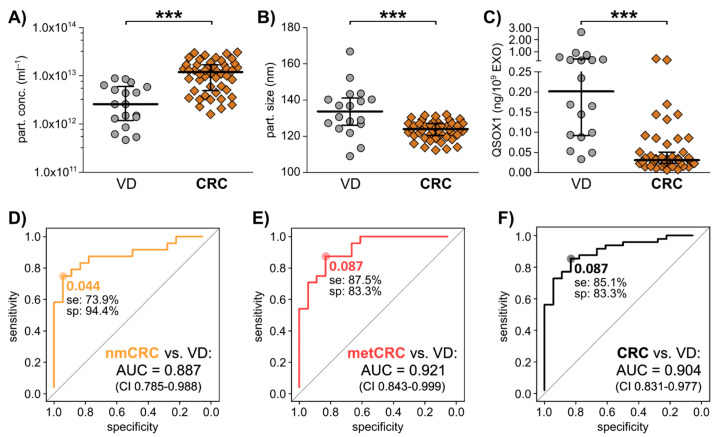
The potential of pEXO QSOX1 as early diagnostic biomarker in CRC liquid biopsy. Blood samples of 48 independent CRC patients were collected immediately prior to surgery, and plasma was subjected to EV isolation. 18 VD patients were analysed as control. (**A**) Particle concentration and (**B**) size of isolated plasma EXOs, examined by NTA analysis using ZetaView^®^. (**C**) Exosomal QSOX1 protein content in the two patient groups as measured by ELISA. Mann-Whitney-U test: *** *p* < 0.001. (**D**–**F**) Diagnostic ROC curve analysis of pEXO-QSOX1, comparing non-metastatic CRC (nmCRC, **D**), metastatic CRC (metCRC, **E**) and all CRC patients (**F**) vs. VD patients. Optimal cut-off values reflecting highest sensitivity (se) and specificity (sp) were calculated by Youden Index.

As we identified a significant negative correlation of QSOX1 to CRC patient-derived pEXOs in the discovery cohort (Figure 2B,C), an independent cohort of pEXO samples comprising 48 patients with CRC (24 non-metastatic, 24 metastatic) was used to examine the pEXO content of QSOX1. Blood samples were collected prior to tumour resection. Patients undergoing surgery due to vascular disease (VD, *n* = 18) that had not been diagnosed with malignant diseases (determined by clinical history and preoperative imaging) were included as controls. Both patient groups underwent surgical intervention at the same hospital, ensuring equal blood collection, processing, storage and documentation measures. Clinicopathologic details of the patients included in this cohort are summarised in Table S4. pEXOs were isolated and characterised by NTA, revealing an overall median particle size of 125.4 nm (107.2–166.8 nm) with an overall median concentration of 1.14 × 10^12^ particles/mL (4.70 × 10^10^–4.40 × 10^12^ particles/mL) in the CRC group, which were significantly different to the EXO concentrations (median 2.6 × 10^11^ particles/mL, Figure 5A) and the particle size (median 133.8, Figure 5B) in the VD controls.

After vesicular protein analysis using ELISA, we calculated the target protein content relative to EXO count and observed a significantly decreased abundance of exosomal QSOX1 in CRC patients compared to VD group (Figure 5C). Correlation of the QSOX1 levels to TNM staging revealed that CRC patients with T stage 0–2 already showed significant decreased QSOX1 levels in their pEXOs compared to VD with a trend towards increasing pEXO QSOX1 content upon progression to T3–4 (Figure S7A). No significant associations of QSOX1 to other clinical parameters (gender, age, primary tumour site, nodal stage, metastasis status, pretreatment) were found (Figure S7).

Diagnostic ROC curve analysis comparing non-metastatic CRC (nmCRC) patients, metastatic CRC (metCRC) patients or all CRC patient with VD returned AUC values of 0.887 (confidence interval 0.785–0.988), 0.921 (confidence interval 0.842–0.999) and 0.904 (confidence interval 0.831–0.977), respectively (Figure 5D–F). Further, an optimal cut-off of 0.087 ng QSOX1/10^9^ pEXOs could differentiate metCRC and overall CRC patients from VD with a sensitivity of 87.5% and 85.1% (Figure 5E,F).

These results propose QSOX1 as a non-invasive, early CRC biomarker in liquid biopsy based on pEXO protein content and warrants further investigations of different patient cohorts necessary to validate the applicability of this marker in a clinical setting.

## 4. Discussion

In order to identify novel non-invasive biomarkers for early CRC diagnosis, markers associated with cells of the TME are of special interest. Beginning from earliest tumour onset, surrounding cells alter their phenotype in response to the malignant transformation. One of the most prominent conversions herein is the activation of NFs to CAFs, displaying specific and differential protein expression. As each cell secretes EXOs displaying cell of origin characteristics, and these EXOs can be isolated from all body fluids, such fibroblast activity markers might also be reflected in blood-derived EXOs. Hence, such markers could enable the non-invasive diagnosis of malignancies at early disease onset. In this study, a comparison of the EXO-associated secretome of patient-matched NFs and CAFs revealed the existence of activity status related protein patterns in fibroblast-derived EXOs. Three selected proteins (QSOX1, THBS1 and EDIL3) were detected in CRC patient pEXOs (shown in Figure 2) and might be useful candidates in early cancer diagnostics engaging non-invasive liquid biopsy.

Several studies have already shown the potential applicability of TME-related markers in tumour diagnostics. For example, differential gene expression patterns between complete tumour stroma, irrespective of the individual cell types, and normal adjacent tissue displayed a promising platform for prediction of disease outcome in breast cancer [10]. A similar study in CRC underlined the prognostic relevance of the TME, as aggressive tumours with shortened recurrence-free survival showed induced expression of genes associated with activated stroma [56]. Other analyses focusing on CAFs revealed the prognostic power of fibroblast activation-related markers in CRC and lung cancer [11,12], without claiming diagnostic relevance. In order to unravel new potential biomarkers specifically for early diagnosis of CRC, we established primary cultures of tumour-educated CAFs and their matched NFs from a total of 13 patients to investigate differential protein expression and the correlating secreted EXO proteome.

Characterisation of the basal protein expression in the established fibroblast cell lines revealed an overall increased αSMA and CAV1 expression combined with an overall reduced expression of CD90 in CAFs compared to respective NFs. Interestingly, several NFs also expressed notable levels of αSMA, similar to results reported by Berdiel-Acer and colleagues [12]. This highlights the necessity to investigate additional markers in order to clarify the unique activity status of fibroblasts. FSP1 and FAPα showed heterogeneous expression patterns throughout the established cell lines. These two markers are often expressed strongly in the invasion front of tumours [57]. Hence, the localisation of the individual tumour pieces relative to the whole tumour mass might affect the expression strength of these two markers in CRC-derived fibroblasts in vitro. Meanwhile, CAV1 is induced in the tumour stroma of several cancer types [58,59] and showed significantly induced expression in the established primary colon CAFs. CD90/Thy1, which is strongly reduced in liver myofibroblasts upon fibrosis [60], showed a significantly reduced expression in the majority of colon CAFs compared to NFs, further underlining their activated myofibroblast phenotype.

Following the successful characterisation of tumour tissue-derived fibroblasts as activated myofibroblasts in contrast to NFs, we intended to unravel CAF-specific markers in fibroblast-derived EXOs. In contrast to other EV subtypes like microvesicles or apoptotic bodies, the biogenesis of EXOs is generally accepted to engage specific sorting mechanisms that co-evolve during cellular changes like tumourigenesis [61]. Similarly, EXO loading processes are expected to adapt during fibroblast activation, resulting in an altered EXO cargo. Mass spectrometry analysis revealed 11 proteins of differential abundance in CAF-EXOs compared to NF-EXOs in the discovery cohort (patients 1–3). Gene set enrichment analysis reflected an association of 7 proteins to EMT and adhesion, of whom six showed high significance and differential expression in the performed mass spectrometric analysis. These proteins (THBS1, FBLN1, EDIL3, QSOX1, ACTN4, MMP3) also displayed differential mRNA expression in CRC compared to healthy intestine in the CRC TCGA dataset [41], and are implicated in tumour growth [54,62,63,64,65,66,67,68,69,70] and metastasis regulation [55,63,65,66,67,71,72,73,74,75,76,77]. Regulatory effects on angiogenesis have been shown for FBLN1 [63], QSOX1 [78], THBS1 [79,80] and EDIL3 [69]. In addition, an association with fibroblasts/CAFs [80,81,82,83,84,85,86,87] and EVs [64,66,72,76,88,89,90,91,92] was described for each protein. In tumour tissue, the expression of all six markers was shown to be of prognostic value in several tumour types [71,72,77,93,94,95]. The current knowledge on the identified fibroblast-derived, EV-associated, EMT- and adhesion-related proteins is summarised in Table S5.

In general, THBS1 and FBLN1 are extracellular matrix (ECM) proteins implicated in tissue remodeling [68,81]. In addition, THBS1 functions as an endogenous angiogenesis inhibitor. QSOX1 is an oxidase, catalysing disulfide bonds in target proteins [96] known to be implicated in fibroblast growth regulation upon quiescence [97] and is therefore a potential NF marker. EDIL3, also known as developmental endothelial locus 1 (DEL-1), is an integrin ligand implicated in angiogenesis promotion [98] and inflammation inhibition [99]. ACTN4 is an intracellular actin-binding protein implicated in cell motility [100].

Following validation of three markers of interest on exosomal as well as cellular protein levels in patients 1–3 by immunoblotting, their abundance was investigated in 10 additional NF/CAF pairs. Overall statistical evaluation returned a significant reduction of QSOX1 in CAFs compared to NFs on exosomal and cellular protein expression level, and significantly induced expression of EDIL3 in whole cell lysate. In agreement of these results, Herrera et al. identified induced expression of EDIL3 in CAFs with a low promigratory potential compared to NFs, while QSOX1 was reduced on the cellular level [85]. Interestingly, in CAFs with a high promigratory potential, the proteins were inversely regulated, indicating that a differential regulation of these markers in fibroblasts is related to differential tumour progression profiles. THBS1 did not show a differential regulation in CAFs compared to NFs in our analyses, neither in whole cells nor in derived EXO-associated protein. EDIL3 did not reach significance in the exosomal protein immunoblot analysis, which could be associated to its function as an immune system modulator.

Since biomarker evaluation in tissue can only be performed after tissue biopsy or resection, in the next step, patient-matched blood samples were investigated to examine the non-invasive diagnostic potential of the biomarkers identified. The CAF-EXO specific proteins THBS1 and EDIL3 identified in vitro showed increased abundance in the pEXO fraction, the NF-EXO specific protein QSOX1 showed markedly reduced abundance in pEXOs as compared to wP and edP. This underlined the specificity of QSOX1 to the pEXO fraction in patient-matched plasma samples. In addition, EDIL3 and QSOX1 abundance was significantly reduced in malignant vs. adjacent non-malignant colorectal tissue. Together, these results underlined the potential of QSOX1 as a biomarker for CRC and justified further examination of pEXOs in an independent test cohort.

Hence, pEXOs of 18 VD and 48 CRC patients were subjected to QSOX1 protein cargo analysis using ELISA. Healthy blood donors were not included in this discovery cohort, as in the majority of cases no clinical history and follow up is available and fasting was not ensured. Our investigation revealed significantly decreased levels of QSOX1 in pEXOs derived from CRC patients compared to VD and a diagnostic potential based on ROC curve analysis.

This study has some limitations. First, our primary fibroblast cell lines derive from CRC patients that underwent surgical resection and might be influenced by the different mutational composition of the primary tumours and also the therapeutic choice. Studies have indicated that fibroblasts can be affected by chemotherapy [101,102,103,104]. However, no significant associations between treatment-naïve and pre-treated fibroblasts were observed in this study, which might be explained by the small cohort of patients or the time frame between receiving neoadjuvant therapy and resection of the tumour. To evaluate whether the biomarkers identified here are influenced by treatment choice and response requires an increased cohort size.

Further research is warranted to investigate the behaviour of pEXO-associated QSOX1 during tumour progression and therapy response. This could be achieved by using longitudinal plasma samples. Secondly, the test cohort assessed for pEXO-associated QSOX1 in CRC patients comprised only 66 patients. Evaluating this marker in a larger patient cohort, including healthy patients and patients with benign colorectal diseases like diverticulitis or early colorectal adenoma, is necessary to confirm the reliability of the pEXO marker QSOX1 in the non-invasive detection of malignant transformations. This will form part of our future work and is also necessary to rule out a potential VD-associated increase of pEXO QSOX1 levels. Third, to investigate whether QSOX1 is specific to CRC or can be found in other cancers, blood samples from other tumour entities will need to be assessed. Furthermore, in vitro experiments need to be considered to investigate the distinct function of QSOX1 in CRC.

## 5. Conclusions

The identified candidate liquid biopsy CRC biomarker QSOX1 might be a useful indicator for malignant transformations in the large intestine. Decreased pEXO levels of QSOX1 could become a useful clinical criterion to forward concerned patients to further diagnostics. Thereby, the progression of pre-malignant lesions and early low-grade adenocarcinomas into advanced tumours could be prevented by early surgical intervention during colonoscopy, promoting a markedly decreased mortality due to CRC.

## Data Availability

The data presented in this study are available in Ganig, Baenke et al., Proteomic analyses of fibroblast- and serum-derived exosomes identify QSOX1 as a marker for non-invasive detection of colorectal cancer. The mass spectrometry data will be openly available in Vesiclepedia.

## Supplementary Materials

The following are available online at https://www.mdpi.com/2072-6694/13/6/1351/s1, Figure S1: IHC analysis in QuPath®, Figure S2: Clinicopathologic timelines of patients 1–13, Figure S3: Number of identified proteins in mass spectrometry, Figure S4: mRNA expression of six identified proteins of interest in TCGA CRC, provided by Oncomine, Figure S5. Fibroblast activity marker expression in an independent validation cohort, Figure S6: In vivo marker expression in patient-matched healthy and malignant colon tissue, Figure S7: Extended patient data correlation on pEXO QSOX1 levels, Figure S8: Uncropped Western Blot Figures, Table S1: Clinicopathologic information on patients 1–13, Table S2: Mass spectrometric data and statistical analysis results for pat. 1–3 NF- and CAF-derived EXOs in biological triplicates, Table S3: Gene set enrichment analysis of 11 proteins differentially abundant in NF- vs. CAF-EXOs in patients 1–3, Table S4: Patient characteristics of an independent pEXO test cohort, Table S5. Overview on current knowledge on fibroblast activity related EXO proteins identified by mass spectrometry.
