# Supplementary material for: Proteomic Analyses of Fibroblast- and Serum-Derived Exosomes Identify QSOX1 as a Marker for Non-invasive Detection of Colorectal Cancer
Source: Cancers (Basel). 2021 Mar 17;13(6):1351. doi: 10.3390/cancers13061351 (PMC8002505; doi:10.3390/cancers13061351)
Supplement: Supplementary file 1 [file cancers-13-01351-s001.zip › cancers-1104651-Supplementary Figures S1-S7.pdf]

Supplementary Material: Proteomic Analyses of Fibroblast and Serum Derived Exosomes Identify QSOX1 as A Marker for Non-Invasive Detection of Colorectal Cancer

Nicole Ganig, Franziska Baenke, May-Linn Thepkaysone, Kuailu Lin, Venkatesh S. Rao, Fang Cheng Wong, Heike Polster, Martin Schneider, Dominic Helm, Mathieu Pecqueux, Adrian M. Seifert, Lena Seifert, Jürgen Weitz, Nuh N. Rahbari and Christoph Kahlert

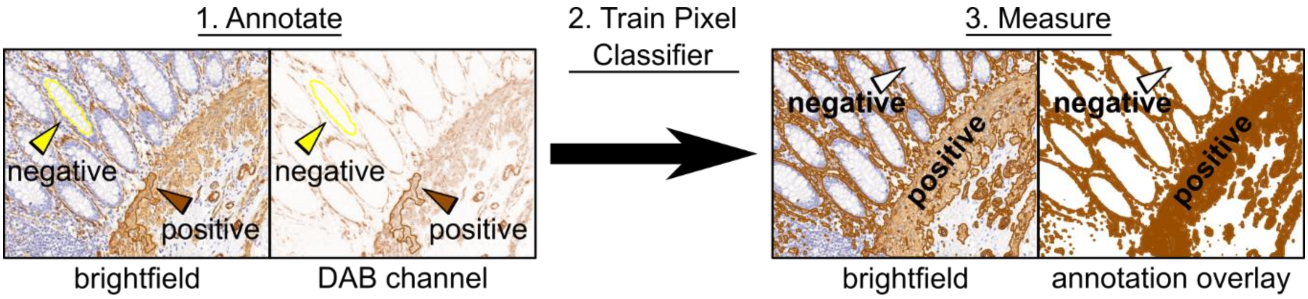

Figure S1. IHC analysis in QuPath®.

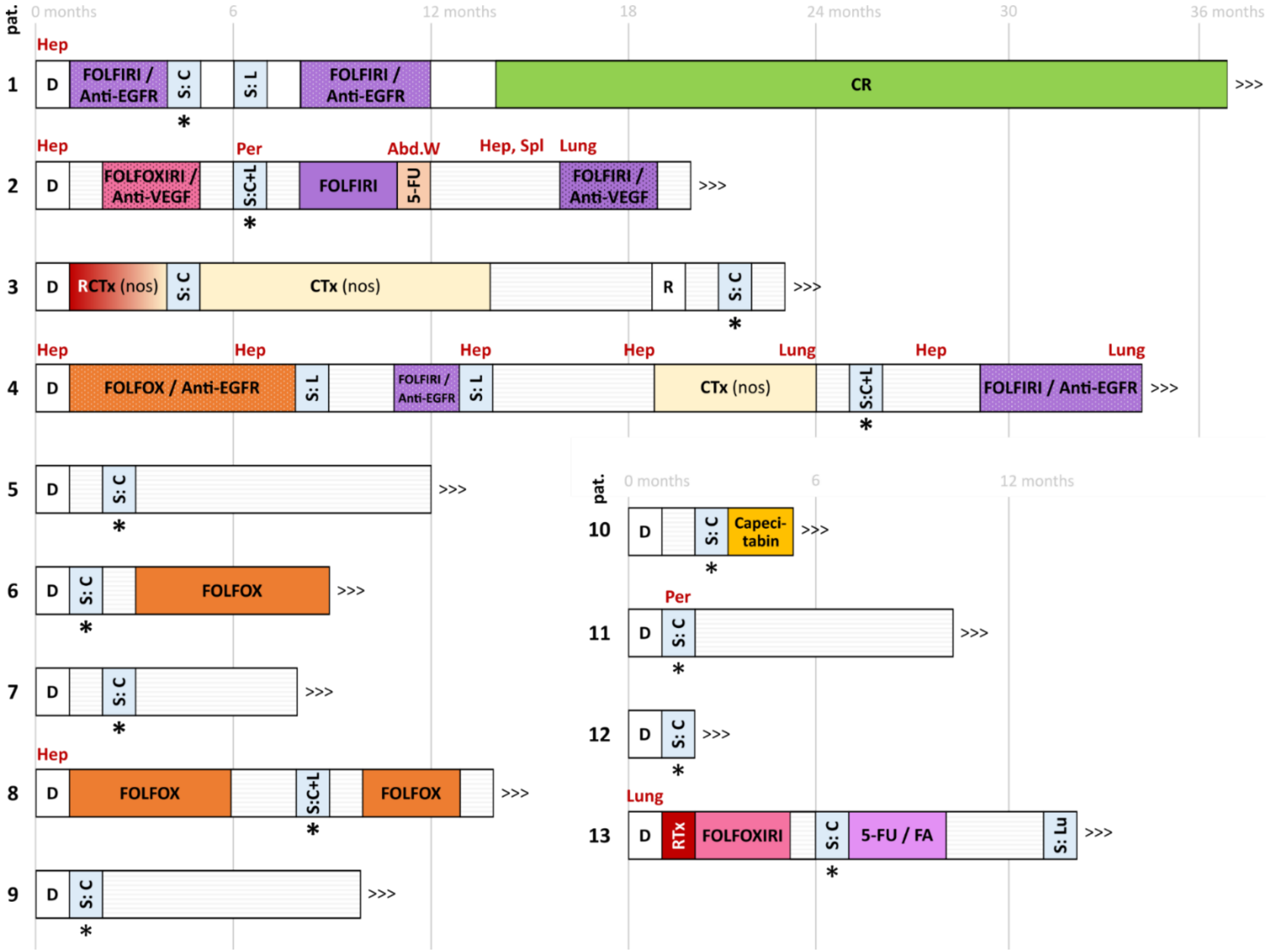

Figure S2. Clinicopathologic timelines of patients 1–13. Timelines reflecting disease progression and applied therapy approaches for each patient, with asterisks (*) highlighting the time point of tissue collection. Locations of distant metastasis diagnosed over time are indicated in red letters. Abbreviations: 5-FU: 5-Fluorouracil; Abd.W: abdominal wall; C: colon;

CR: complete remission; CTx: chemotherapy; D: diagnosis; EGFR: epidermal growth factor receptor; FOL: folinic acid; FOLFIRI: FOL/5-FU/Irinotecan combination therapy; FOLFOX: FOL/5-FU/Oxaliplatin combination therapy; FOLFOXIRI: FOL/5-FU/Oxaliplatin/Irinotecan combination therapy; Hep: hepatic; L: liver; Lu: lung; nos: no other specified; pat.: patient; Per: peritoneum; R: local recidive; RCTx: radiochemotherapy; RTx: radiotherapy; S: surgery; Spl: spleen; VEGF: vascular endothelial growth factor.

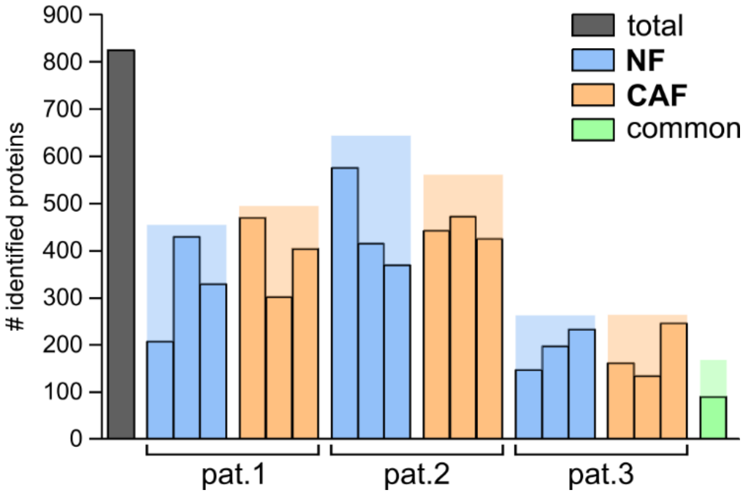

Figure S3. Number of identified proteins in mass spectrometry. Primary fibroblast-derived EXOs were subjected to proteomic analysis ($n = 3$). The overall number of identified proteins per sample (proteins identified in at least one replicate) is indicated in faint colors. Abbreviations: CAF: cancer-associated fibroblast; EXO: exosome; NF: normal fibroblast; pat.: patient.

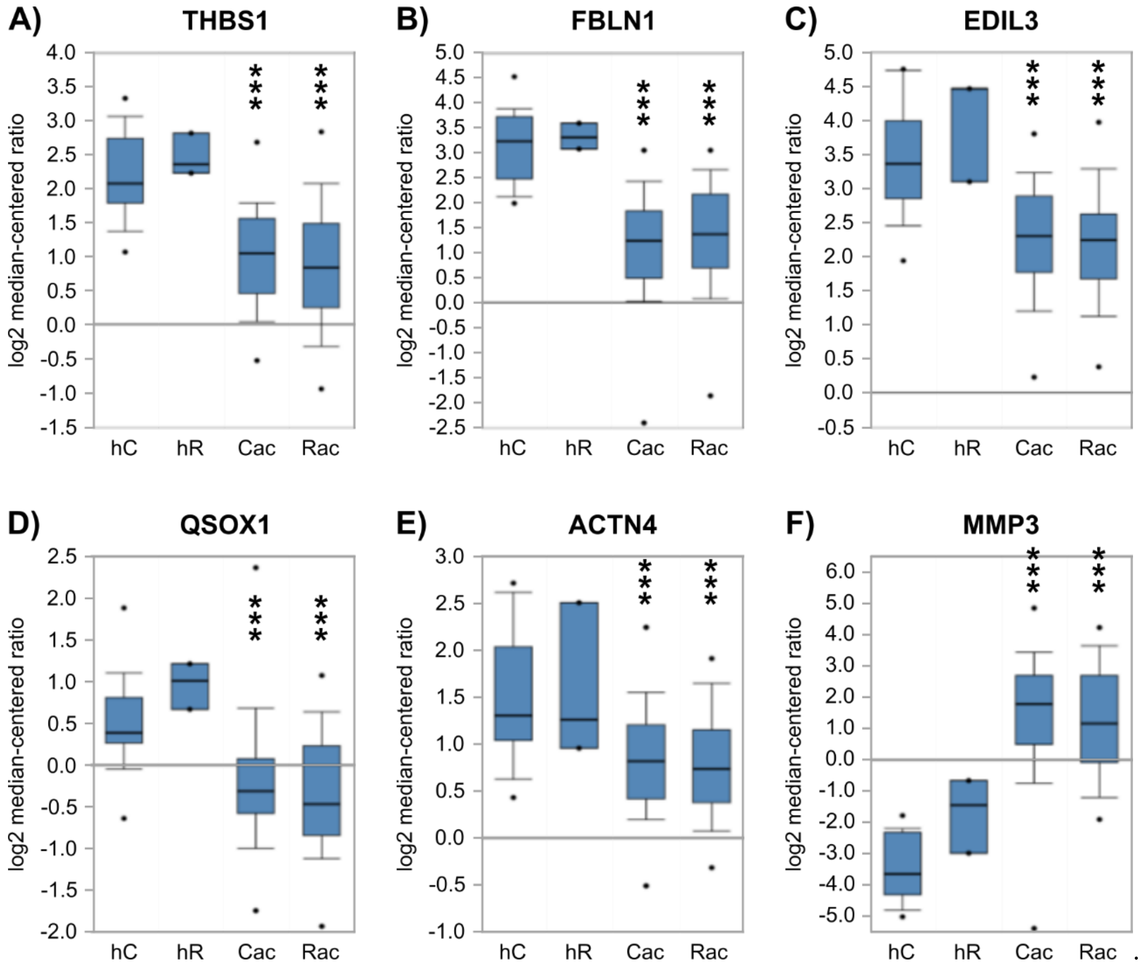

Figure S4. mRNA expression of six identified proteins of interest in TCGA CRC, provided by Oncomine**.** Columns display healthy colon (hC, n = 19), healthy rectum (hR, n = 3), colon adenocarcinoma (Cac, n = 101) and rectum adenocarcinoma (Rac, n = 60). Statistical differences as compared to healthy samples (hC and hR): *** $p < 0.001$. (**A)** Thrombospondin 1 (THBS1, reporter A_23_P206210). (**B)** Fibulin 1 (FBLN1, reporter A_23_P211630). (**C)** EGF-like repeats and discoidin domains 3 (EDIL3, reporter A_23_P401606). (**D)** Quiescin sulfhydryl oxidase 1 (QSOX1, reporter A_23_P12463). (**E)** Actinin α4 (ACTN4, reporter A_23_P315241). (**F)** Matrix metallopeptidase 3 (MMP3, reporter A_23_P161696). Abbreviations: Cac: colon adenocarcinoma; hC: healthy colon; hR: healthy rectum; Rac: rectum adenocarcinoma.

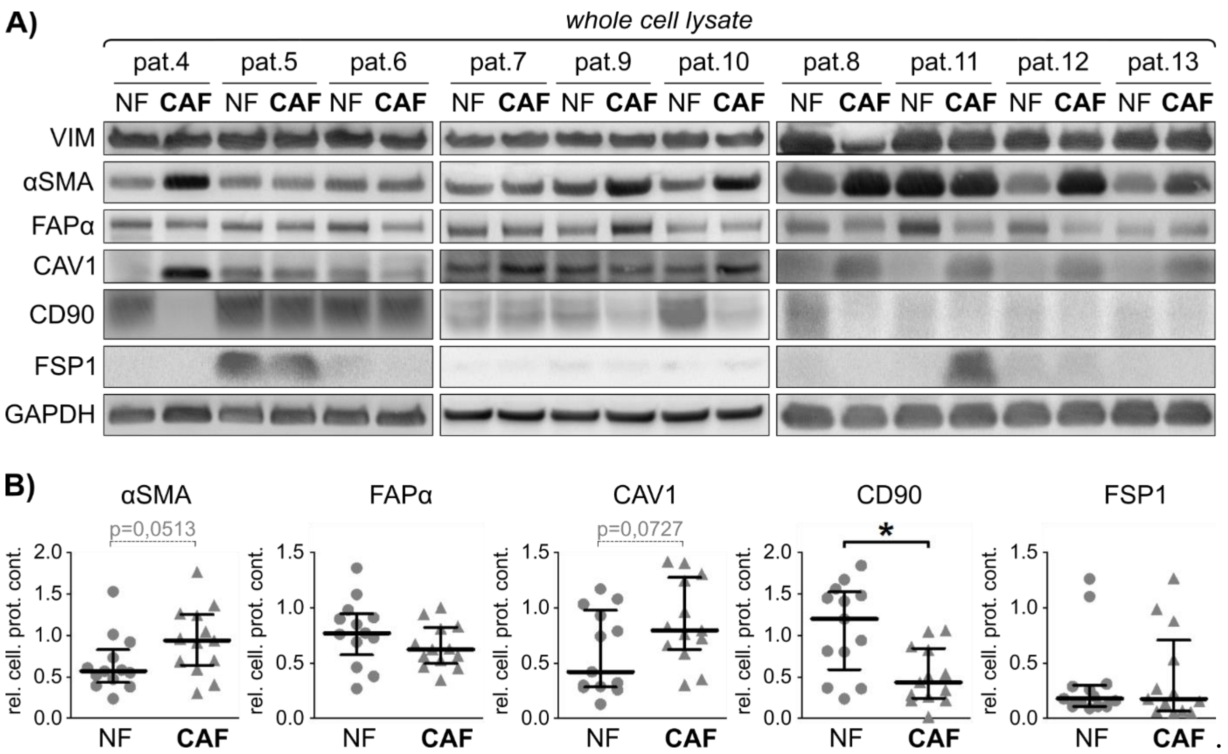

Figure S5. Fibroblast activity marker expression in an independent validation cohort. Twenty fibroblast cell lines derived from 10 CRC patients were subjected to cellular protein isolation and subjected to Immunoblot. **(A)** Immunoblot analysis of vimentin (VIM), α-smooth-muscle actin (αSMA), fibroblast activation protein α (FAPα), caveolin 1 (CAV1), cluster of differentiation 90 (CD90) and fibroblast-specific protein 1 (FSP1) in primary fibroblasts, including GAPDH as loading control. **(B)** Graphical analysis of immunoblots shown in (**A**) and (Figure. 1A) using ImageJ, relative to GAPDH. Mann-Whitney-U test: * $p < 0{,}05$. Abbreviations: CAF: cancer-associated fibroblast; CRC: colorectal cancer; GAPDH: glyceraldehyde 3 phosphate dehydrogenase; NF: normal fibroblast; pat.: patient; rel. cell. prot. cont.: relative cellular protein content.

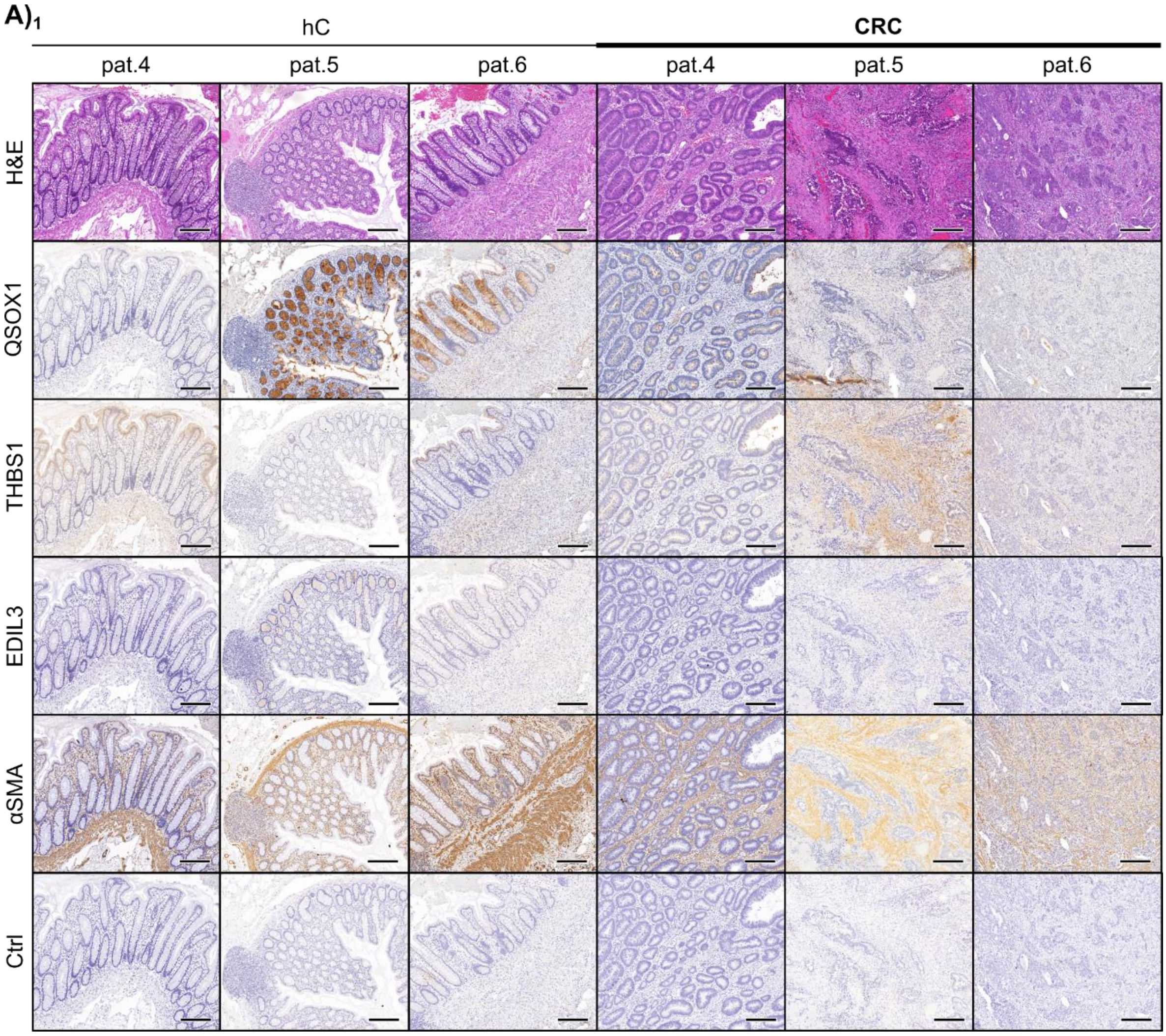

A)1
hC
CRC
pat.4
pat.5
pat.6
pat.4
pat.5
pat.6
H&E
QSOX1
THBS1
EDIL3
αSMA
Ctrl

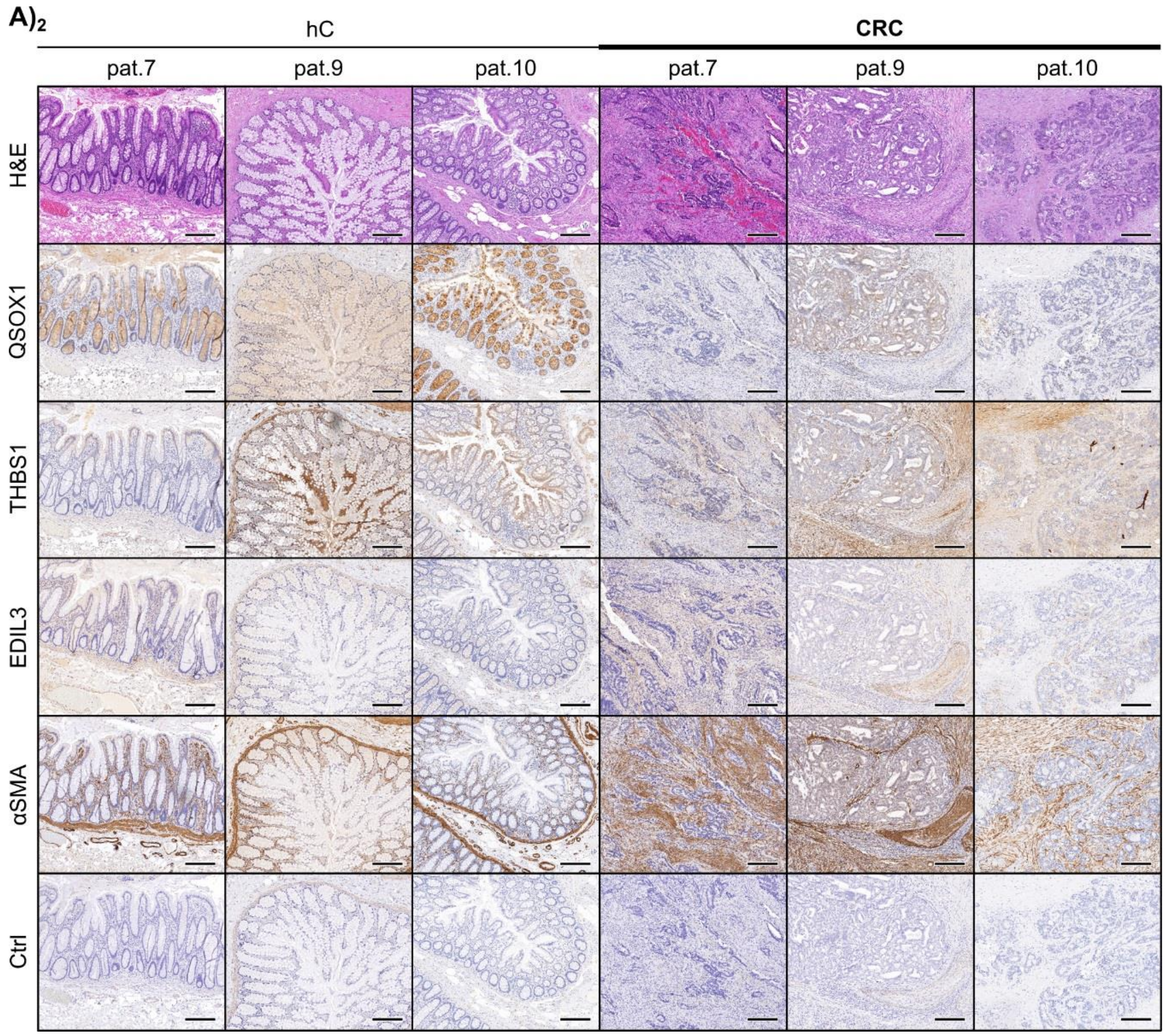
A)2
hC
CRC
pat.7
pat.9
pat.10
pat.7
pat.9
pat.10
H&E
QSOX1
THBS1
EDIL3
αSMA
Ctrl

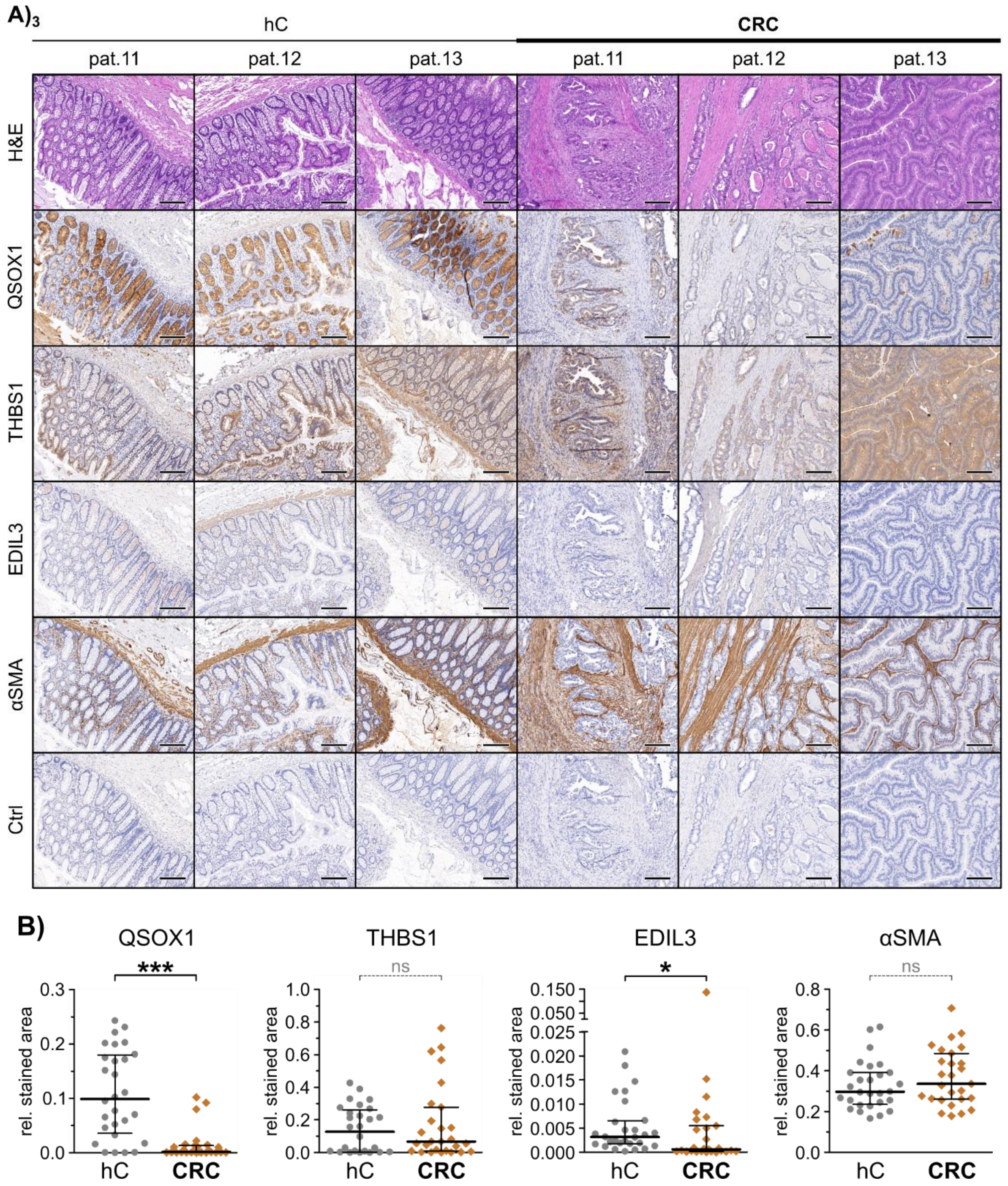

Figure S6. In vivo marker expression in patient-matched healthy and malignant colon tissue. **(A)**1-3 Representative images of paraffin embedded tissue slides of healthy and malignant colon tissue derived from patients 4-7 and 9-13, H&E or immunohistochemically stained for the proteins QSOX1, THBS1, EDIL3, αSMA and IgG control (Ctrl). Scale bars equal 250 µm. **(B)** Graphical IHC staining analysis performed in QuPath. From each patient and tissue, a minimum of three representative areas were subjected to graphical and statistical analysis. Mann-Whitney-U test: *** $p < 0.001$, * $p < 0.05$, ns = not significant. Abbreviations: αSMA: α-smooth-muscle actin; CRC: colorectal cancer; Ctrl: control; EDIL3: EGF-like repeats and discoidin domains 3; H&E: hematoxylin and eosin stain; hC: healthy colon; IgG: immunoglobulin G; IHC: immunohistochemistry; pat.: patient; THBS1: thrombospondin 1, QSOX1: quiescin sulfhydryl oxidase 1.

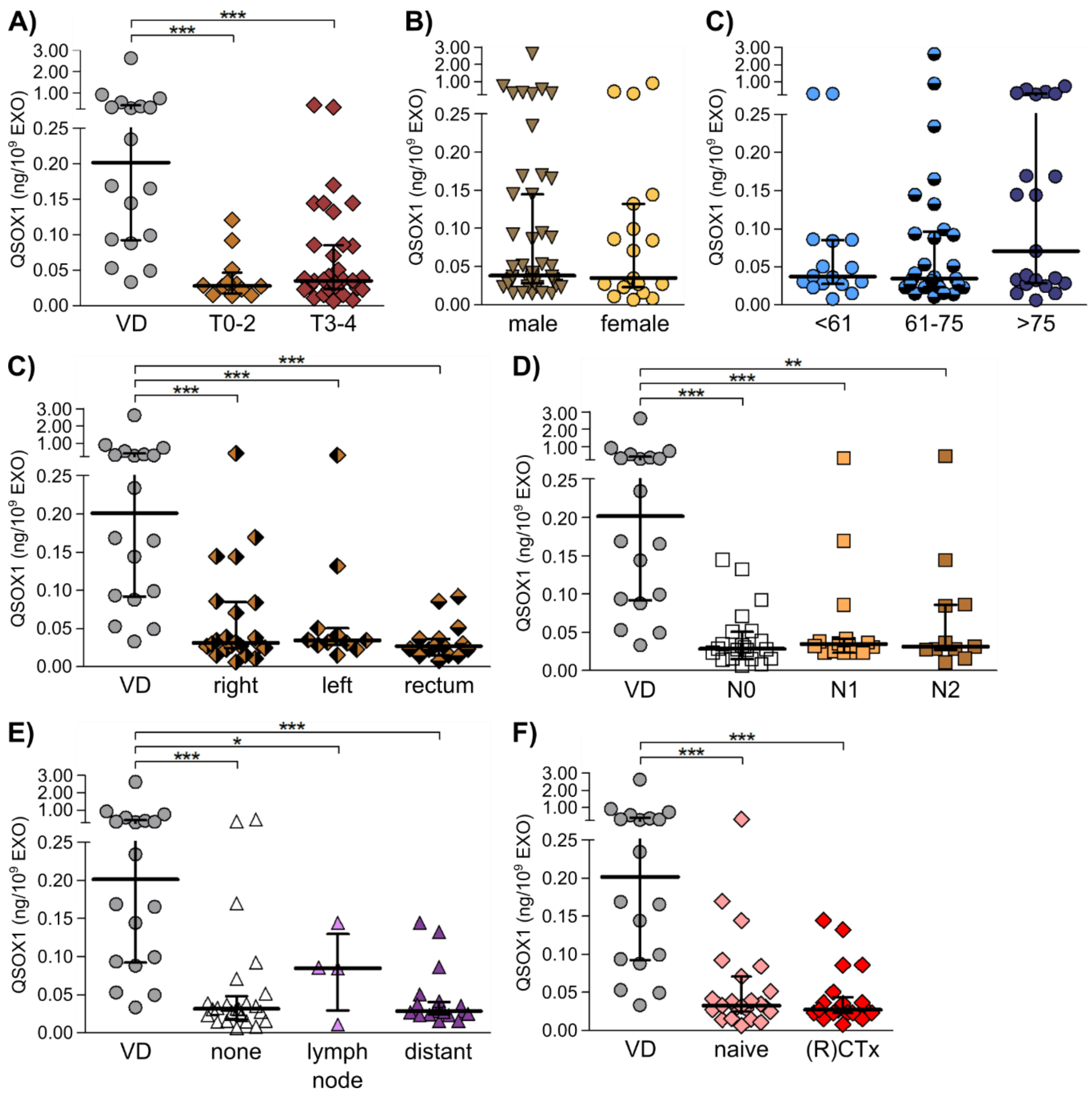

Figure S7. Extended patient data correlation on pEXO QSOX1 levels**.** pEXO levels of QSOX1 depending on T stage **(A)**, gender **(B)**, age at time point of surgery and blood sampling **(C)**, primary colorectal tumour site **(D)**, N stage **(E)**, metastasis status **(F)** and pretreatment **(G)**. Mann-Whitney-U test: * $p < 0.05$, ** $p < 0.01$, *** $p < 0.001$. Abbreviations: pEXO: plasma-derived exosomes; EXO: exosome; QSOX1: quiescin sulfhydryl oxidase 1; (R)CTx: (radio-)chemotherapy; VD: vascular disease
