# Supplementary material for: Proteomic Analyses of Fibroblast- and Serum-Derived Exosomes Identify QSOX1 as a Marker for Non-invasive Detection of Colorectal Cancer
Source: Cancers (Basel). 2021 Mar 17;13(6):1351. doi: 10.3390/cancers13061351 (PMC8002505; doi:10.3390/cancers13061351)
Supplement: Supplementary file 1 [file cancers-13-01351-s001.zip › cancers-1104651-Supplementary Figures S8.pdf]

Figure 1 A)

lanes:
1: fibroblast cellular protein mix
2: pat.1 NF cellular protein
3: pat.1 CAF cellular protein
4: pat.2 NF cellular protein
5: pat.2 CAF cellular protein
6: pat.3 NF cellular protein
7: pat.3 CAF cellular protein
M: Precision Plus Protein All Blue

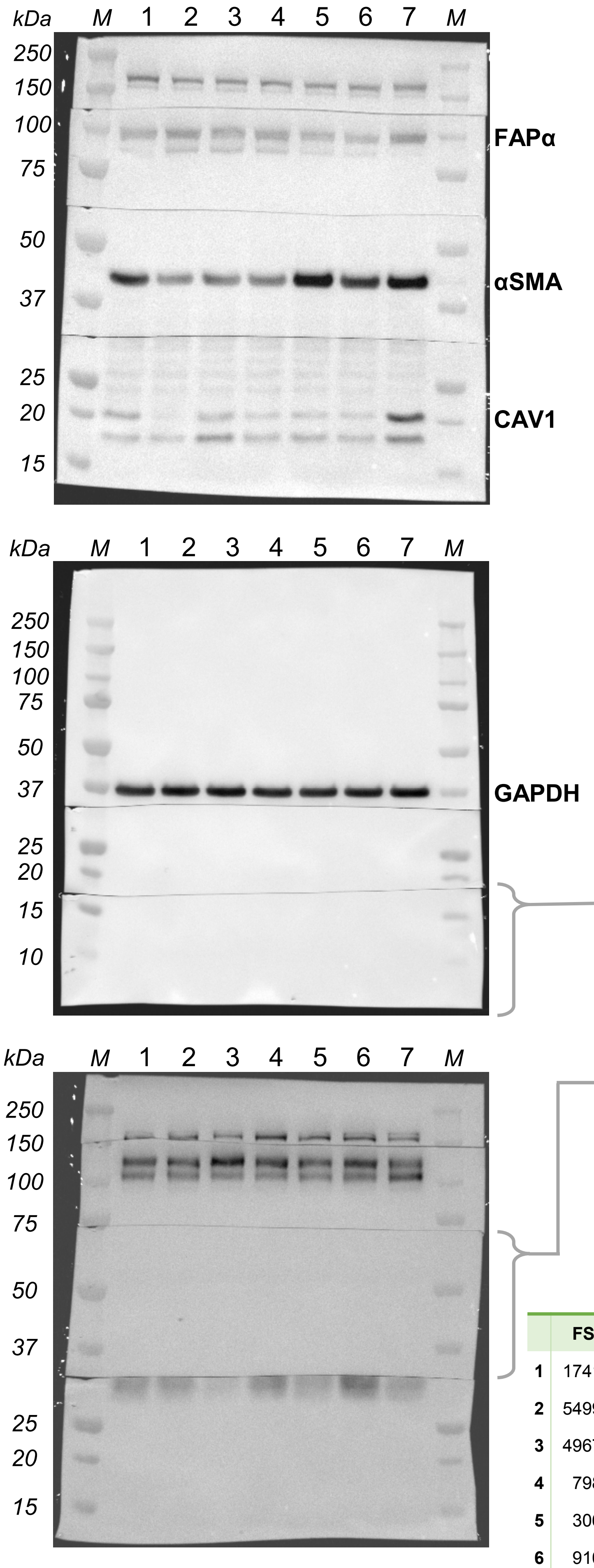

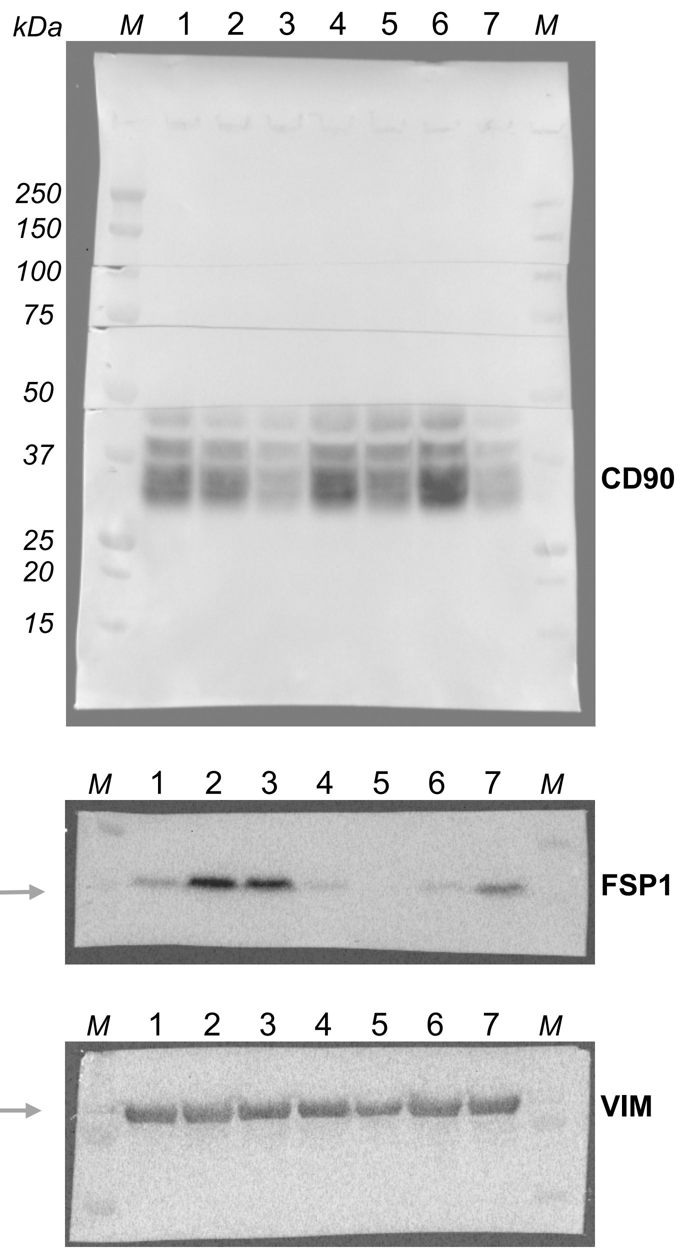

	FSP1	FAPα	αSMA	CAV1	VIM	CD90	GAPDH
**1**	17416.12	33000.17	34617.75	24446.05	53841.48	18438.81	47265.22
**2**	54992.07	37837.63	12908.80	7154.27	45472.82	19977.59	49806.17
**3**	49676.72	30070.92	16186.63	18939.53	49094.31	11070.33	50214.24
**4**	7985.31	34758.46	17255.97	11749.34	47532.77	27419.95	44307.87
**5**	3062.19	21672.63	46067.53	14552.29	31921.87	19970.69	43491.97
**6**	9106.48	17889.05	34322.34	13691.75	49671.14	30294.37	46150.51
**7**	26844.72	39595.95	45872.24	47146.65	48800.43	9914.40	50155.82

Figure 1 D), part 1

lanes:

1: pat.1 NF exosomal protein, n1
2: pat.1 CAF exosomal protein, n1
3: pat.2 NF exosomal protein, n1
4: pat.2 CAF exosomal protein, n1
5: pat.3 NF exosomal protein, n1
6: pat.3 CAF exosomal protein, n1
7: pat.1 NF edp-cM protein, n1
8: pat.1 CAF edp-cM protein, n1
9: pat.2 NF edp-cM protein, n1
10: pat.2 CAF edp-cM protein, n1
11: pat.3 NF edp-cM protein, n1
12: pat.3 CAF edp-cM protein, n1
M: *Precision Plus Protein All Blue*

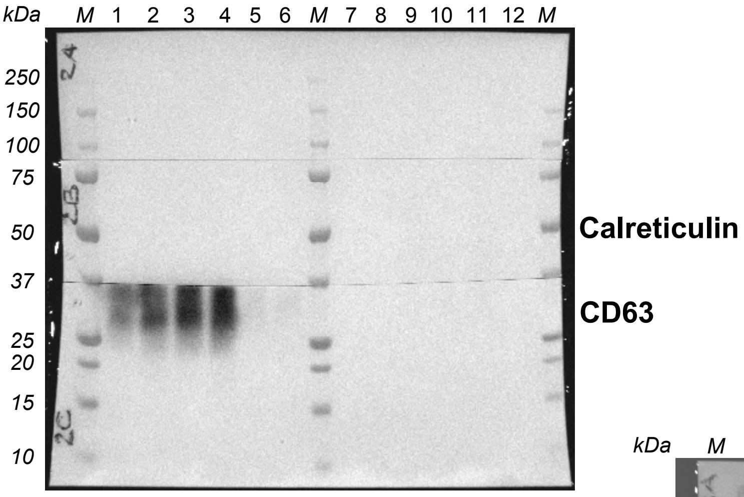

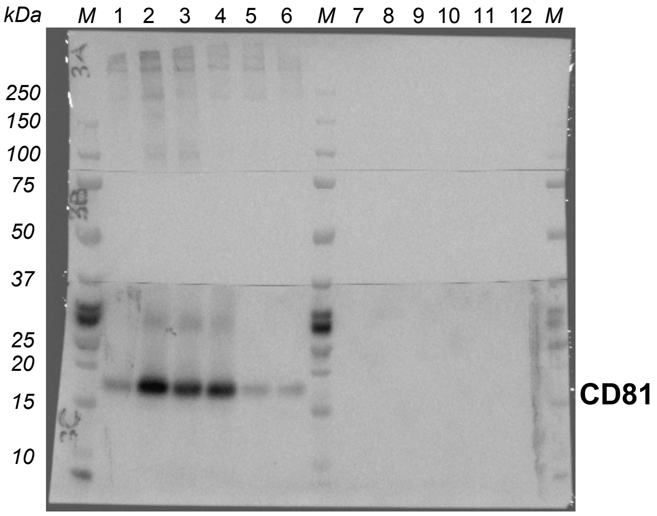

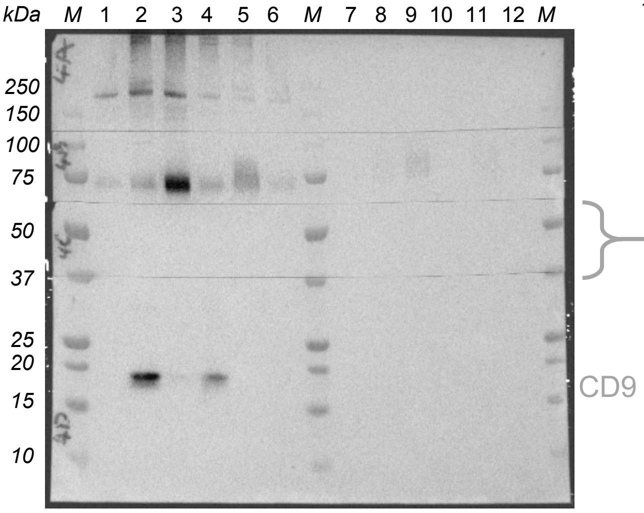

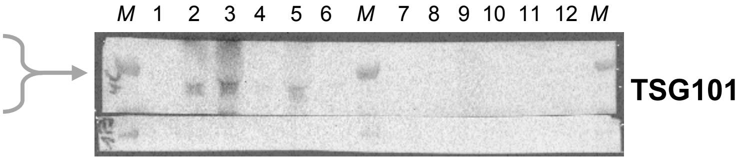

	CD63	CD81	TSG101	Calreticulin
**1**	36959.83	25056.20	10934.61	30480.20
**2**	56581.73	66384.32	30710.61	43454.20
**3**	61904.02	52673.61	50975.61	36627.20
**4**	60266.73	49457.90	11763.61	20173.20
**5**	7935.42	12190.08	23401.61	16389.20
**6**	5752.54	10128.78	9051.61	20737.20
**7**	1232.66	756.57	5617.03	16256.90
**8**	1035.88	1197.54	7360.32	16602.61
**9**	1826.90	944.42	13578.32	22883.90
**10**	2350.20	1396.54	8574.61	22122.61
**11**	2421.25	2032.83	8932.32	16680.90
**12**	81468.07	19710.78	9808.32	7615.32

Figure 1 D), part 2

Figure 1 F), part 1

lanes:

1: pat.1 NF exosomal protein, n2
2: pat.1 CAF exosomal protein, n2
3: pat.2 NF exosomal protein, n2
4: pat.2 CAF exosomal protein, n2
5: pat.3 NF exosomal protein, n2
6: pat.3 CAF exosomal protein, n2
7: pat.1 NF exosomal protein, n3
8: pat.1 CAF exosomal protein, n3
9: pat.2 NF exosomal protein, n3
10: pat.2 CAF exosomal protein, n3
11: pat.3 NF exosomal protein, n3
12: pat.3 CAF exosomal protein, n3

M: Precision Plus Protein All Blue

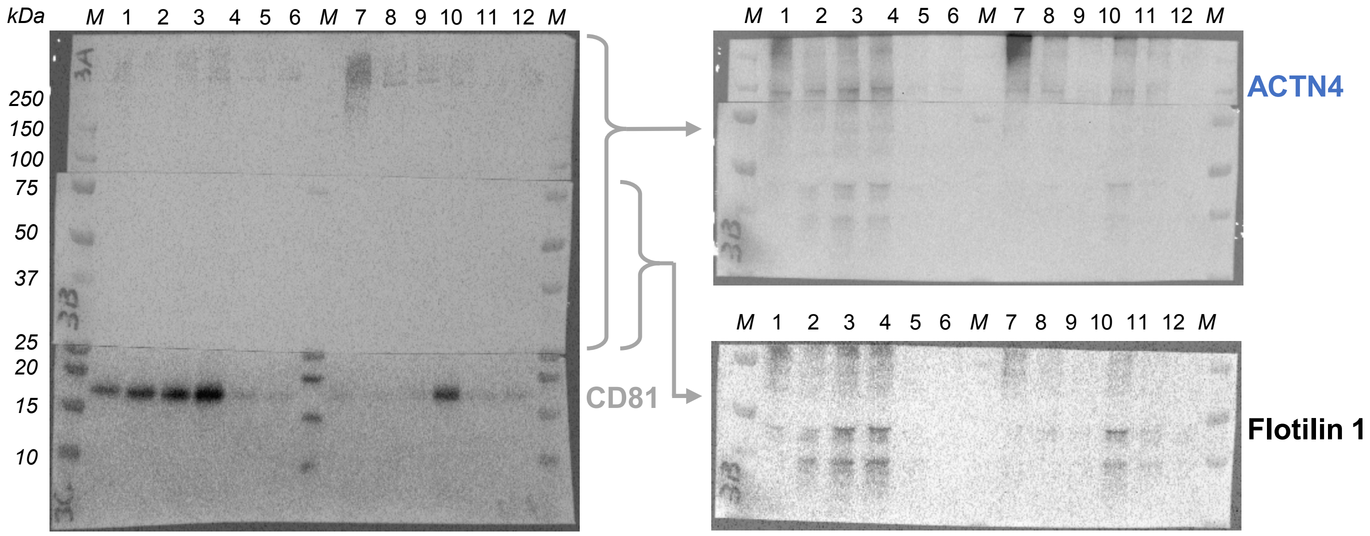

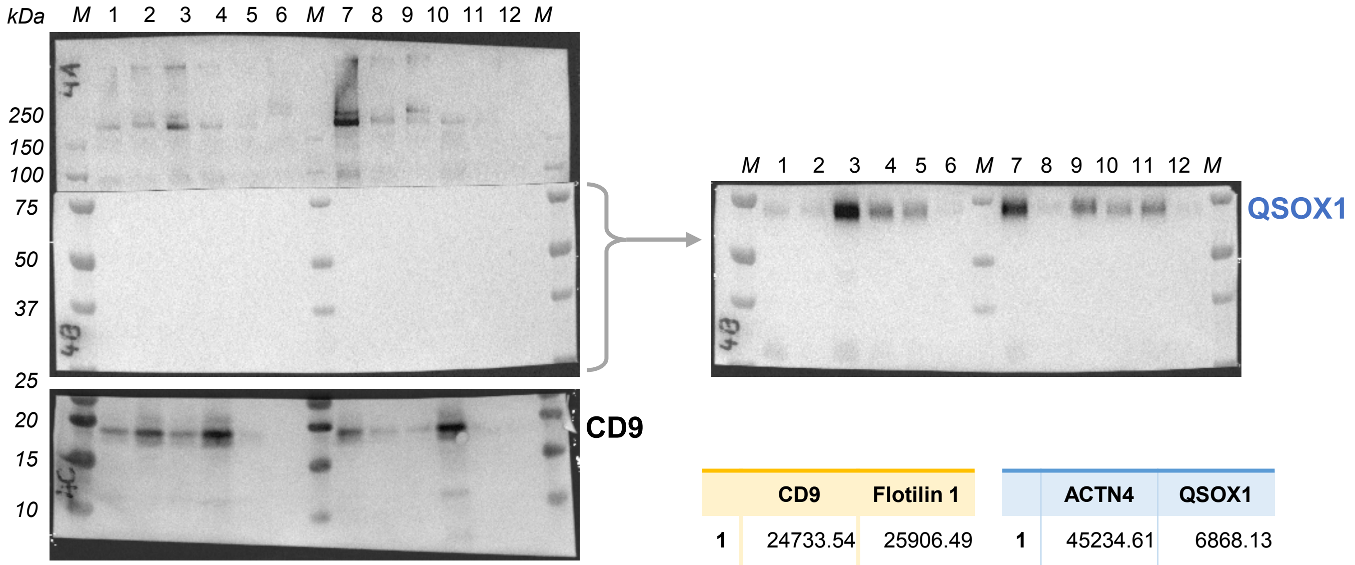

	CD9	Flotilin 1
1	24733.54	25906.49
2	46243.27	28760.49
3	23606.71	48845.49
4	64208.10	47657.78
5	6217.25	11383.78
6	2483.00	7232.49
7	35360.08	19716.49
8	13539.37	23794.78
9	12936.13	22939.20
10	63118.27	47619.49
11	7750.08	35078.78
12	2203.25	30364.29

	ACTN4	QSOX1
1	45234.61	6868.13
2	37775.37	7263.49
3	52371.37	59627.27
4	51383.32	26656.37
5	11247.78	18928.66
6	8383.782	4942.20
7	38609.15	48594.68
8	26345.15	8241.30
9	10051.08	27771.02
10	29161.44	17984.37
11	15656.08	24756.02
12	4393.468	6404.125

Figure 1 F), part 2 **Figure 1 G)**

lanes:
1: pat.1 NF exosomal protein, n2
2: pat.1 CAF exosomal protein, n2
3: pat.2 NF exosomal protein, n2
4: pat.2 CAF exosomal protein, n2
5: pat.3 NF exosomal protein, n2
6: pat.3 CAF exosomal protein, n2
7: pat.1 NF exosomal protein, n3
8: pat.1 CAF exosomal protein, n3
9: pat.2 NF exosomal protein, n3
10: pat.2 CAF exosomal protein, n3
11: pat.3 NF exosomal protein, n3
12: pat.3 CAF exosomal protein, n3
13: pat.1 NF cellular protein
14: pat.1 CAF cellular protein
15: pat.2 NF cellular protein
16: pat.2 CAF cellular protein
17: pat.3 NF cellular protein
18: pat.3 CAF cellular protein
19: fibroblast cellular protein mix
M: Precision Plus Protein All Blue

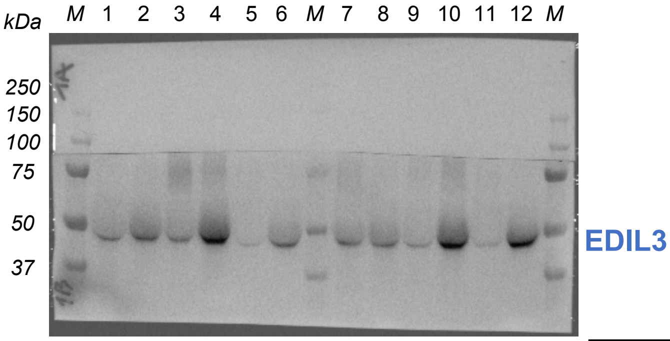

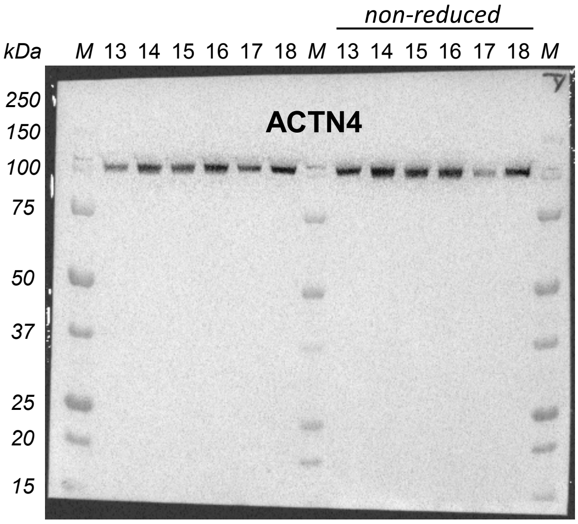

		ACTN4	QSOX1	EDIL3
	13	12133.42	22148.05	2902.65
	14	21267.14	10786.61	2960.33
	15	19221.03	28061.88	2652.70
	16	25268.77	12684.65	2850.64
	17	17051.72	20572.07	3301.12
	18	25616.72	4341.67	3648.91
non-reduced	13	20565.78	26128.49	5792.83
non-reduced	14	26968.32	9281.13	19759.73
non-reduced	15	23201.90	16114.02	13271.95
non-reduced	16	25215.02	5531.25	19753.61
non-reduced	17	9990.83	18899.85	12052.25
non-reduced	18	23960.80	5435.83	26223.49

	EDIL3
1	21502.61
2	35301.44
3	20206.54
4	60444.15
5	3800.71
6	24147.97
7	22275.78
8	26676.90
9	12086.42
10	60866.10
11	9570.18
12	50313.56

	THBS1	GAPDH
1	3026.35	2727.35
2	3376.88	1341.64
3	6248.83	702.52
4	11821.66	1525.64
5	18339.49	1795.93
6	24858.54	2190.35
19	2844.88	4238.35

	THBS1	GAPDH
13	4817.22	27417.27
14	16087.95	29764.49
15	6607.95	32180.73
16	23406.32	30437.49
17	6718.66	31664.08
18	18829.15	28246.02

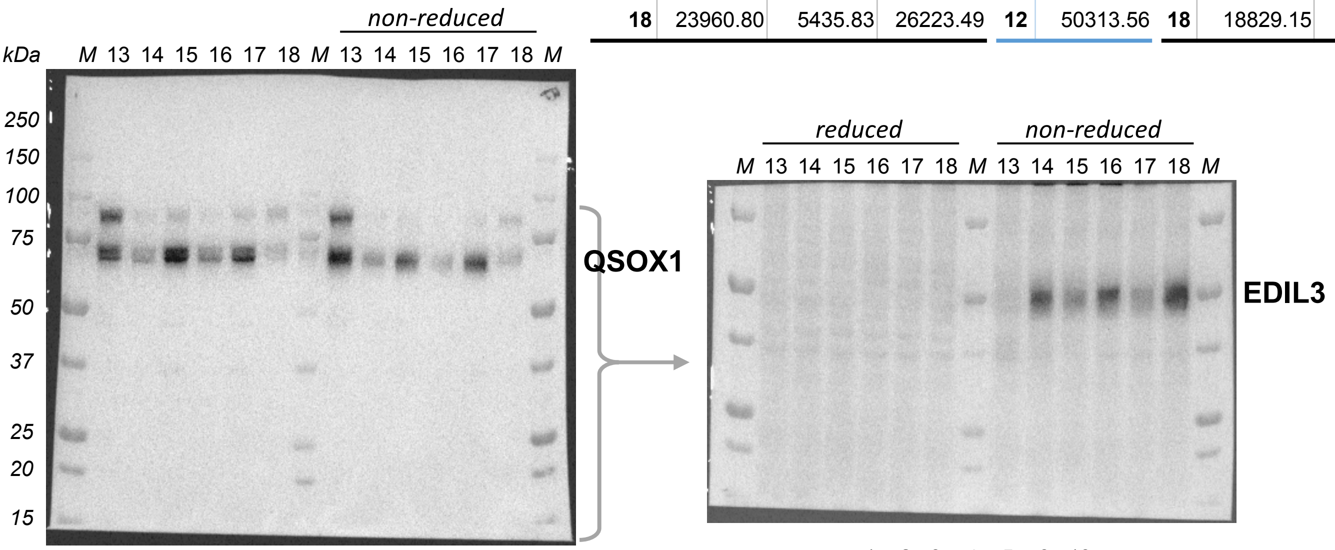

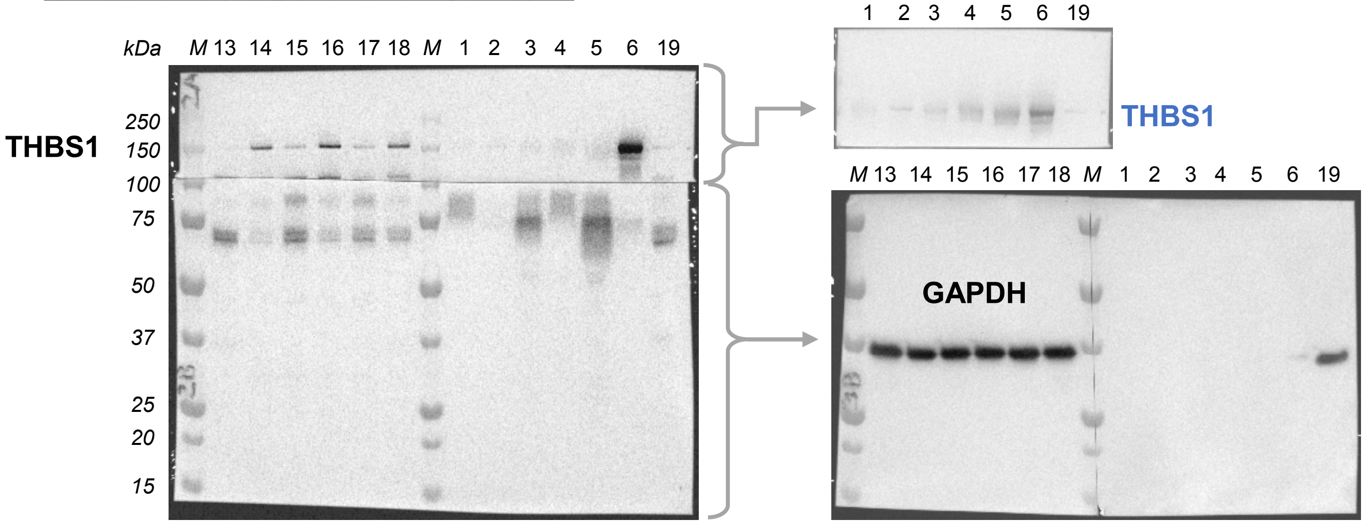

Figure 2 B), part 1

lanes:
1: pat.1 wp protein
2: pat.2 wP protein
3: pat.3 wP protein
4: pEXO protein mix
5: pat.1 pEXO protein
6: pat.2 pEXO protein
7: pat.3 pEXO protein
8: edP protein mix
9: pat.1 edP protein
10: pat.2 edP protein
11: pat.3 edP protein
M: Precision Plus Protein All Blue

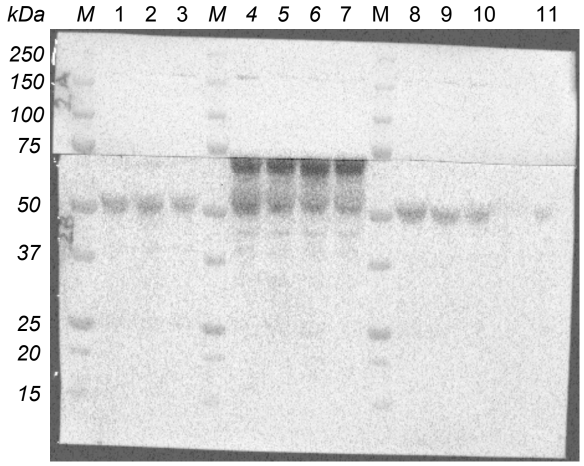

EDIL3

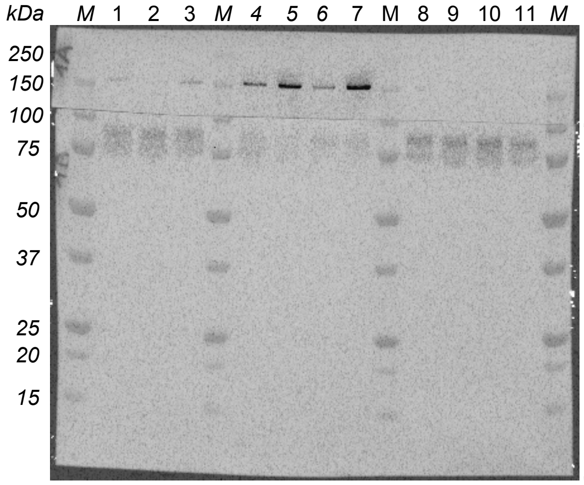

THBS1

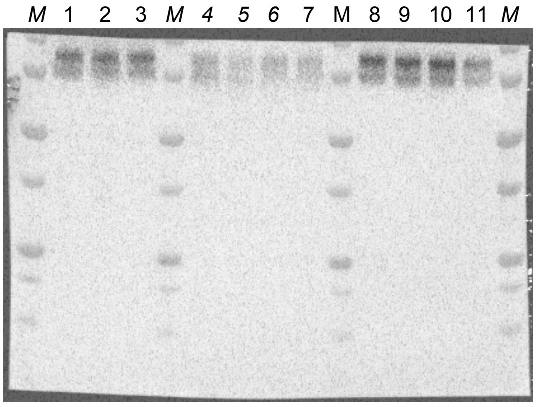

QSOX1

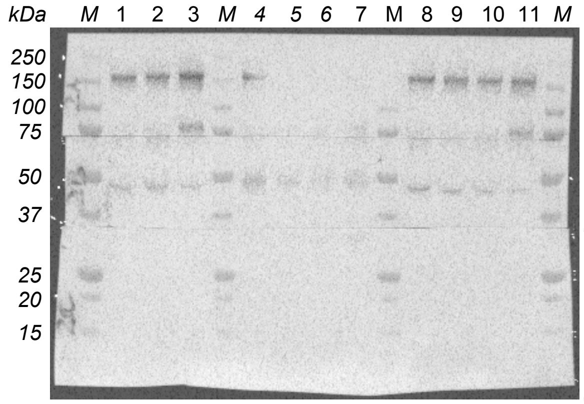

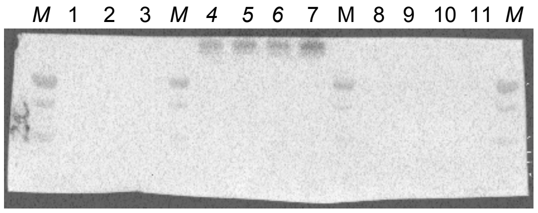

CD9

	THBS1	QSOX1	EDIL3	CD9
**1**	6708.02	49754.85	29675.78	5605.08
**2**	5140.92	51902.73	32339.85	4994.08
**3**	8739.66	47885.02	21788.95	5752.73
**4**	22901.08	29113.90	68299.68	30799.95
**5**	43962.61	16035.54	52831.78	37625.20
**6**	17473.54	20214.20	49948.61	34799.83
**7**	53686.27	20652.44	45097.15	49826.08
**8**	2648.21	53393.73	31840.73	5084.66
**9**	2998.33	57928.49	22585.66	6210.15
**10**	3850.30	57781.90	21973.73	5616.13
**11**	3366.05	34354.02	8892.61	4485.52

Figure 2 B), part 2

lanes:
1: pat.1 wp protein
2: pat.2 wP protein
3: pat.3 wP protein
4: pEXO protein mix
5: pat.1 pEXO protein
6: pat.2 pEXO protein
7: pat.3 pEXO protein
8: edP protein mix
9: pat.1 edP protein
10: pat.2 edP protein
11: pat.3 edP protein
M: Precision Plus Protein All Blue

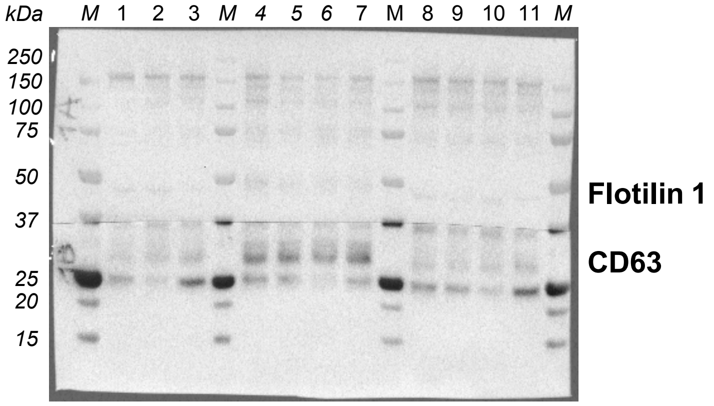

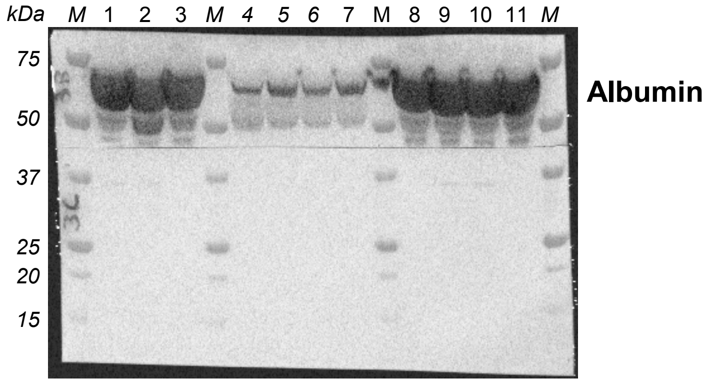

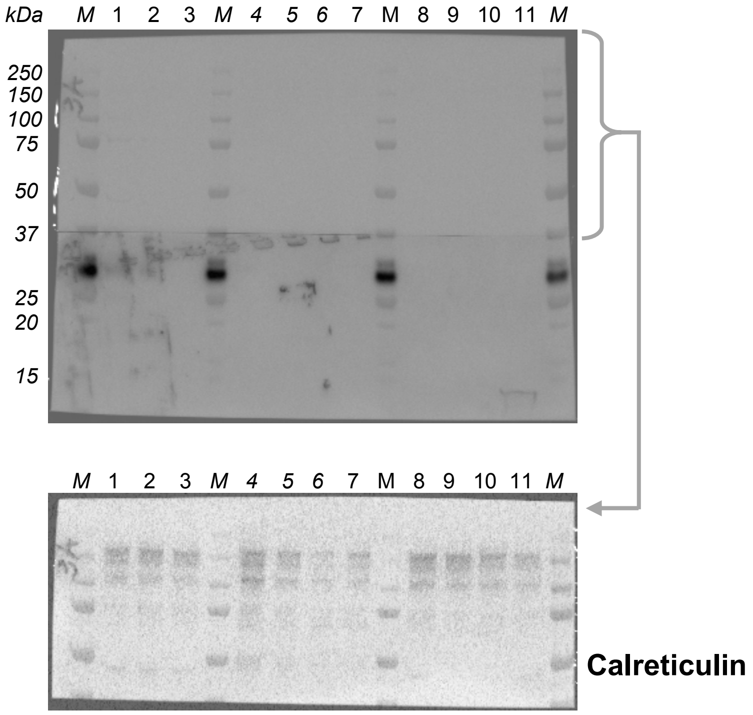

	Flotilin 1	CD63	Albumin	Calreticulin
**1**	17163.18	17000.37	67353.73	31765.25
**2**	19027.44	20010.20	66654.15	31109.61
**3**	12883.25	23884.27	60795.27	27630.20
**4**	49679.90	50990.78	20462.61	46064.66
**5**	26250.20	44864.49	23509.32	33481.66
**6**	25469.37	36617.78	17855.66	31060.37
**7**	28701.90	50824.61	24218.15	29822.73
**8**	23586.49	18740.73	73733.68	18857.97
**9**	16782.49	13585.08	71653.20	7455.66
**10**	21907.66	15512.66	72867.15	14933.37
**11**	15969.08	20166.54	68492.78	24199.83

Figure 4 A)

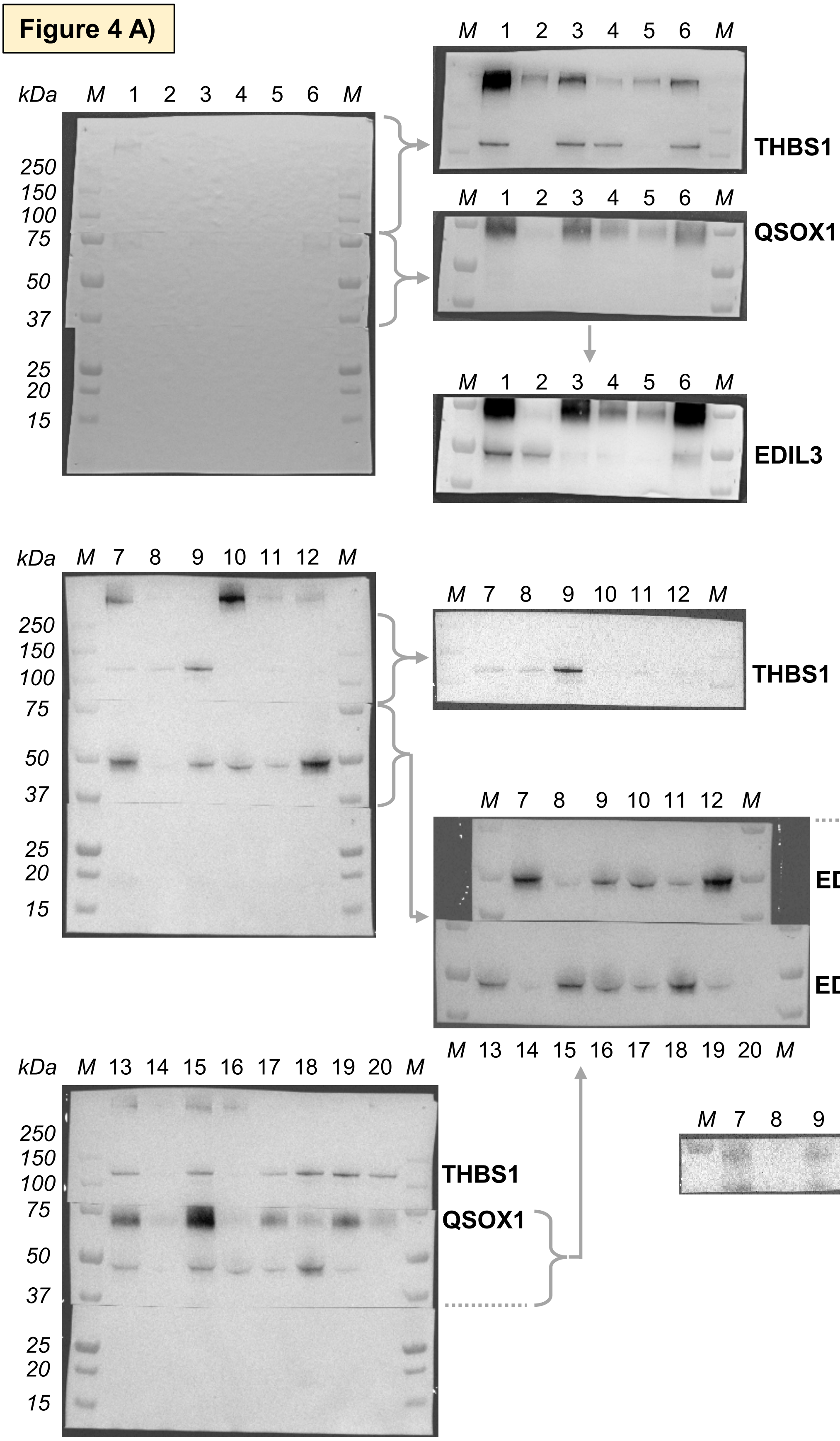

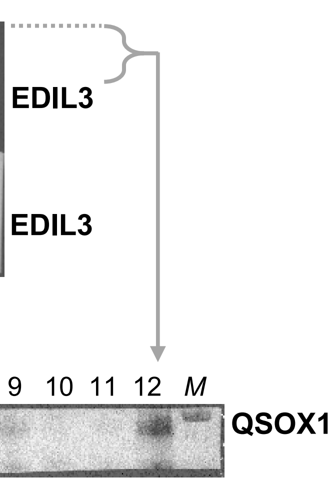

	QSOX1	EDIL3	THBS1
1	81287.18	74723.74	73535.23
2	7716.53	45314.11	8674.45
3	54900.11	12336.72	73953.64
4	23693.79	6384.29	41906.28
5	14853.07	3260.17	2263.90
6	46091.23	36931.28	57996.40
7	45265.74	44017.94	10920.79
8	14153.33	3632.82	12977.92
9	21690.62	20206.97	55197.04
10	15241.62	19460.33	12169.74
11	9013.55	10483.60	15029.38
12	49155.43	60078.57	18937.04
13	39208.55	41231.45	45836.21
14	7353.26	13415.50	13301.04
15	79756.82	51049.04	54570.43
16	6148.53	37262.92	10371.69
17	20373.38	20013.19	21838.02
18	10961.02	71715.16	66204.09
19	32078.45	13884.97	63328.97
20	11573.48	1790.56	36516.09

lanes:

1: pat.4 NF exosomal protein
2: pat.4 CAF exosomal protein
3: pat.5 NF exosomal protein
4: pat.5 CAF exosomal protein
5: pat.6 NF exosomal protein
6: pat.6 CAF exosomal protein

M: Precision Plus Protein All Blue

7: pat.7 NF exosomal protein
8: pat.7 CAF exosomal protein
9: pat.9 NF exosomal protein
10: pat.9 CAF exosomal protein
11: pat.10 NF exosomal protein
12: pat.10 CAF exosomal protein

13: pat.8 NF exosomal protein
14: pat.8 CAF exosomal protein
15: pat.11 NF exosomal protein
16: pat.11 CAF exosomal protein
17: pat.12 NF exosomal protein
18: pat.12 CAF exosomal protein
19: pat.13 NF exosomal protein
20: pat.13 CAF exosomal protein

Figure 4 B), part 1

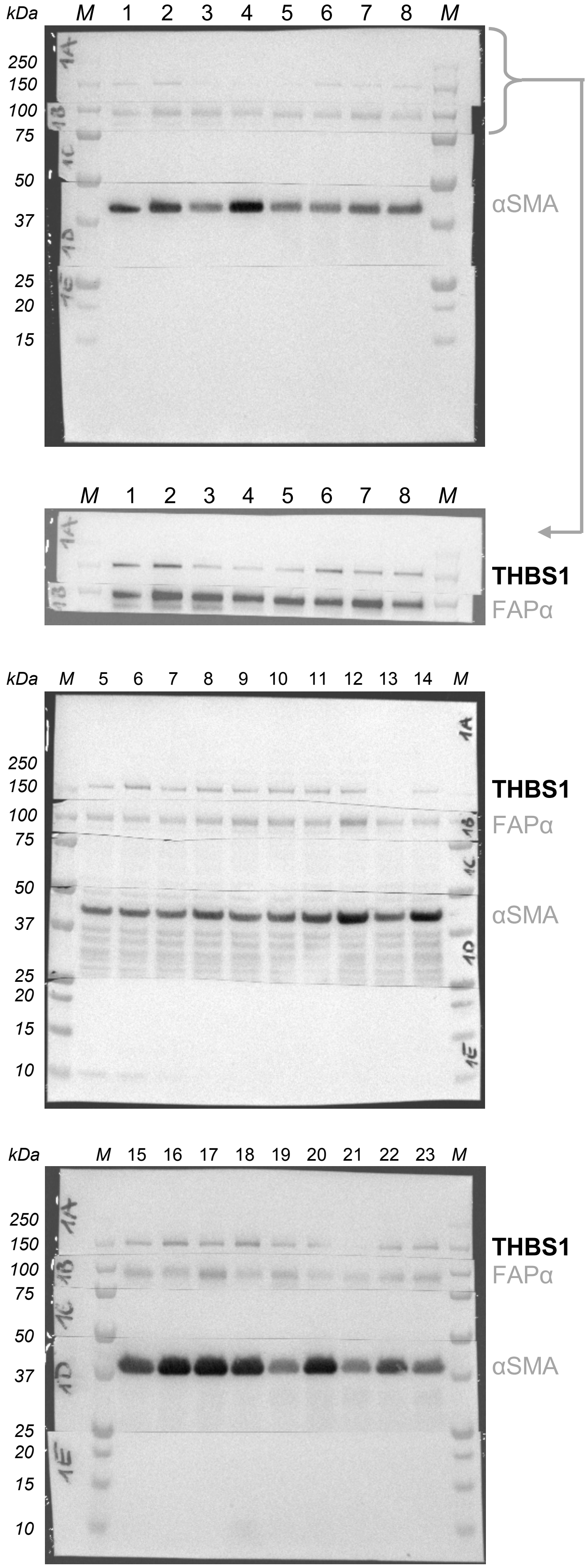

lanes:

1: fibroblast cellular protein mix (1)
2: fibroblast cellular protein mix (2)
3: pat.4 NF cellular protein
4: pat.4 CAF cellular protein
5: pat.5 NF cellular protein
6: pat.5 CAF cellular protein
7: pat.6 NF cellular protein
8: pat.6 CAF cellular protein

M: Precision Plus Protein All Blue

9: pat.7 NF cellular protein
10: pat.7 CAF cellular protein
11: pat.9 NF cellular protein
12: pat.9 CAF cellular protein
13: pat.10 NF cellular protein
14: pat.10 CAF cellular protein

15: pat.8 NF cellular protein
16: pat.8 CAF cellular protein
17: pat.11 NF cellular protein
18: pat.11 CAF cellular protein
19: pat.12 NF cellular protein
20: pat.12 CAF cellular protein
21: pat.13 NF_1 cellular protein
22: pat.13 CAF cellular protein
23: pat.13 NF_2 cellular protein

	THBS1	THBS1
1	38185.80	
2	49143.23	
3	18509.70	
4	10672.02	
5	15050.77	22699.29
6	38096.36	42996.06
7	32380.77	29795.94
8	36080.07	40530.53
9		32386.17
10		37391.82
11		36317.12
12		31221.70
13		4150.22
14		11997.58
15	31011.82	
16	50634.36	
17	39969.53	
18	54562.48	
19	30081.70	
20	18053.65	
21	5370.58	
22	24835.46	
23	31583.11	

Figure 4 B), part 2

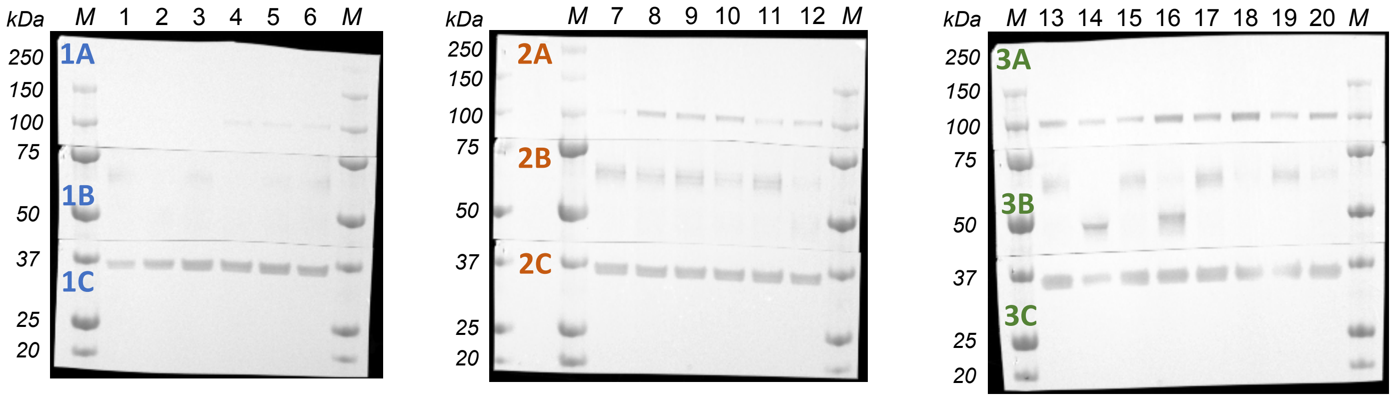

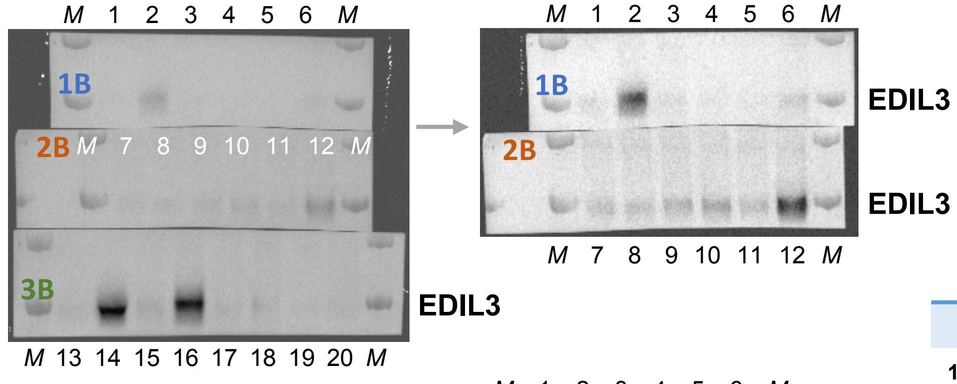

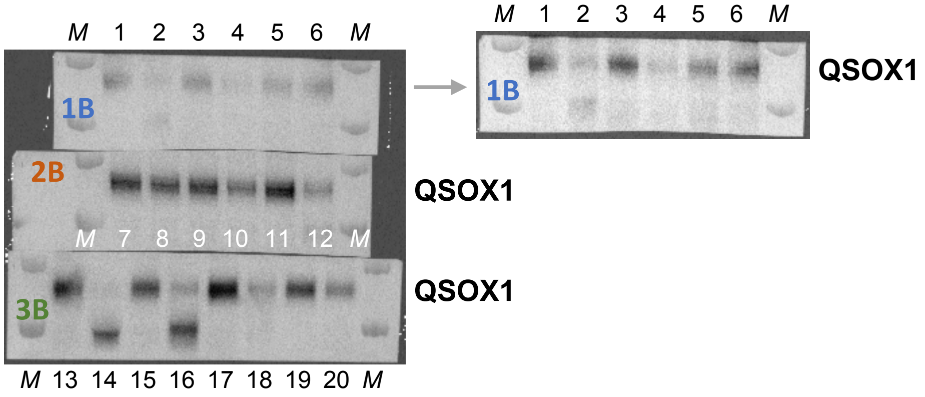

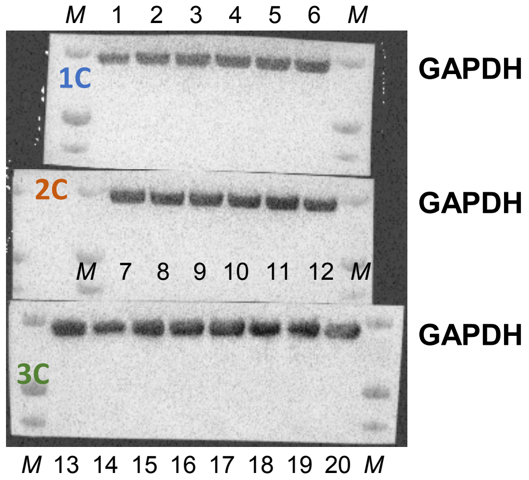

	QSOX1	EDIL3	GAPDH
**1**	40888.07	4908.34	30552.58
**2**	13803.70	43392.43	41245.36
**3**	41819.19	6500.70	46395.07
**4**	10983.82	4094.89	47221.48
**5**	30350.31	4343.41	50312.36
**6**	40343.65	10652.53	51743.65
**7**	34305.82	7847.00	39454.95
**8**	28470.29	8200.70	39600.95
**9**	33249.70	10671.46	40244.41
**10**	19492.87	13549.12	42580.70
**11**	39825.12	8664.05	49127.82
**12**	13003.75	38627.70	39808.29
**13**	50788.29	5922.27	61213.19
**14**	5884.58	55473.89	42527.53
**15**	38987.02	7713.92	57495.41
**16**	16743.70	58817.43	59697.48
**17**	60927.60	5598.05	62346.77
**18**	16548.46	5883.53	56476.87
**19**	40934.48	3123.05	56901.82
**20**	21180.41	5747.53	47688.24

lanes:

1: pat.4 NF cellular protein
2: pat.4 CAF cellular protein
3: pat.5 NF cellular protein
4: pat.5 CAF cellular protein
5: pat.6 NF cellular protein
6: pat.6 CAF cellular protein

7: pat.7 NF cellular protein
8: pat.7 CAF cellular protein
9: pat.9 NF cellular protein
10: pat.9 CAF cellular protein
11: pat.10 NF cellular protein
12: pat.10 CAF cellular protein

13: pat.8 NF cellular protein
14: pat.8 CAF cellular protein
15: pat.11 NF cellular protein
16: pat.11 CAF cellular protein
17: pat.12 NF cellular protein
18: pat.12 CAF cellular protein
19: pat.13 NF cellular protein
20: pat.13 CAF cellular protein

M: Precision Plus Protein All Blue

Figure S5 A), part 1

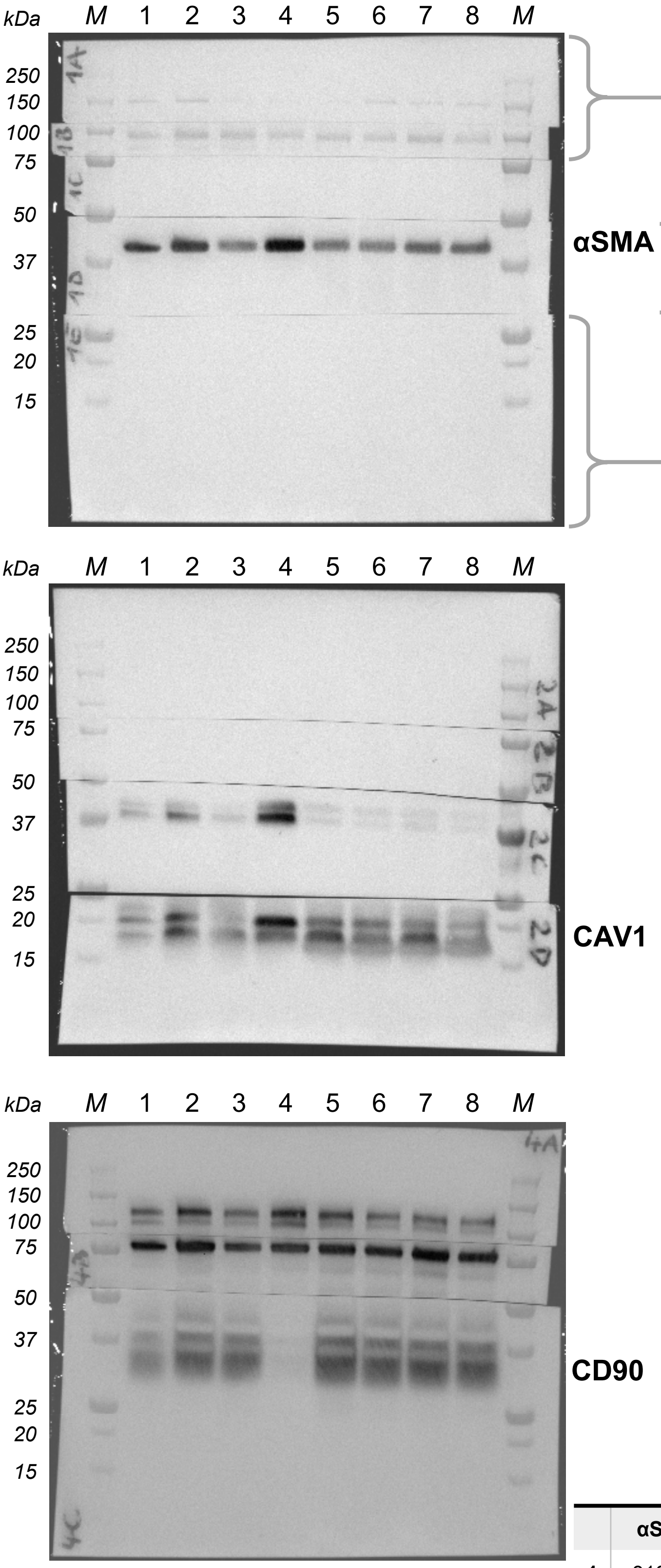

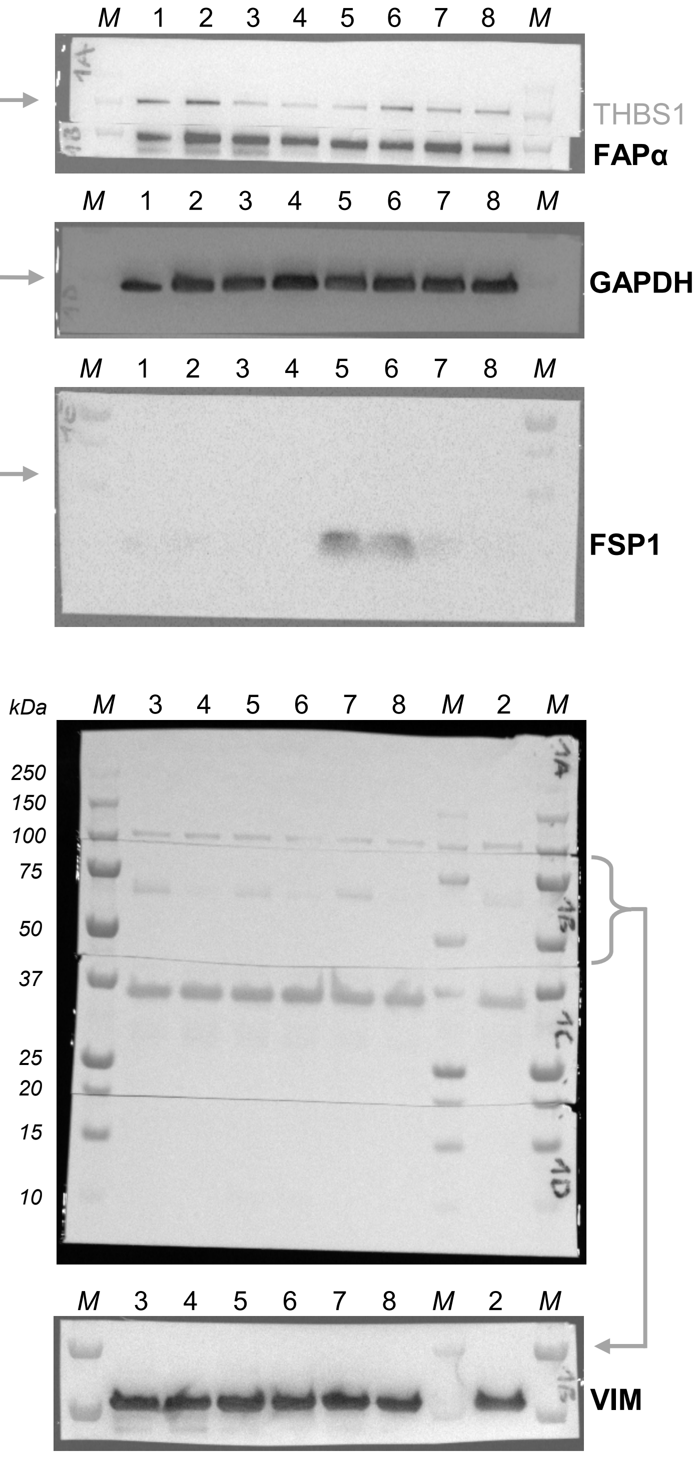

lanes:

1: fibroblast cellular protein mix (1)
2: fibroblast cellular protein mix (2)
3: pat.4 NF cellular protein
4: pat.4 CAF cellular protein
5: pat.5 NF cellular protein
6: pat.5 CAF cellular protein
7: pat.6 NF cellular protein
8: pat.6 CAF cellular protein

M: Precision Plus Protein All Blue

	αSMA	FSP1	FAPα	CAV1	VIM	CD90	GAPDH
**1**	31074.83	5775.24	32411.75	22640.60	-	22827.66	32899.26
**2**	39742.50	7046.48	55195.94	43617.67	53355.38	45769.07	45321.51
**3**	20205.41	3718.36	48276.65	18268.58	62223.48	40567.77	43296.36
**4**	53834.02	3637.67	36672.65	76930.67	62471.70	1053.66	58886.02
**5**	21368.53	51571.02	36742.19	43961.82	63988.65	57631.77	40781.82
**6**	19289.41	41950.65	31961.58	39417.19	59624.87	49969.70	47673.95
**7**	26853.24	14466.43	45790.65	36654.36	65548.65	56107.48	46684.24
**8**	29281.14	6758.72	27748.24	28990.84	53278.65	52520.19	49553.43

Figure S5 A), part 2

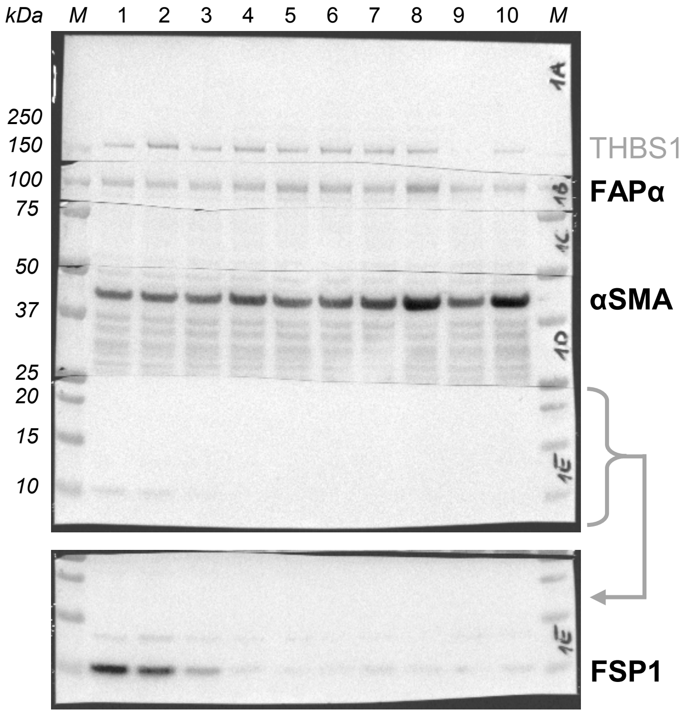

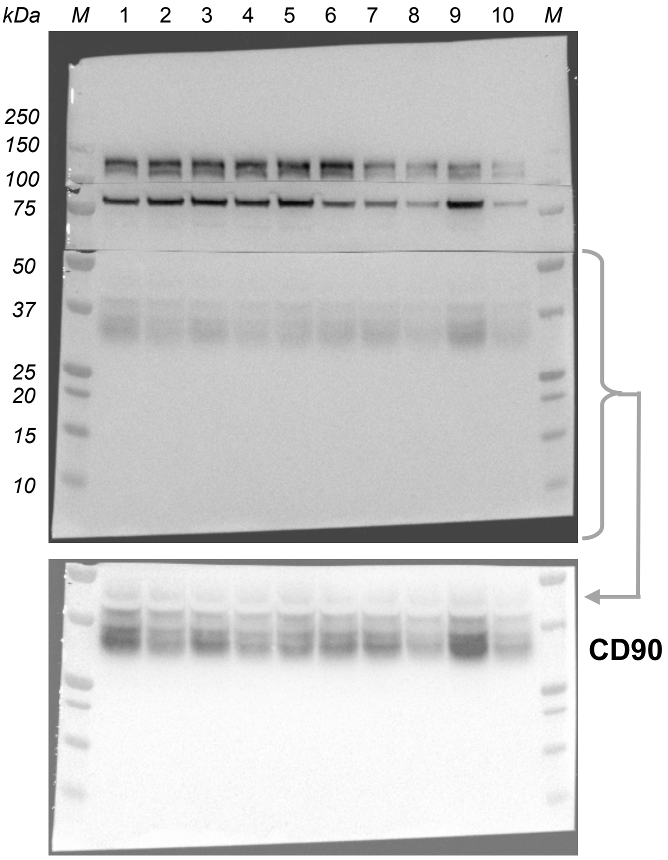

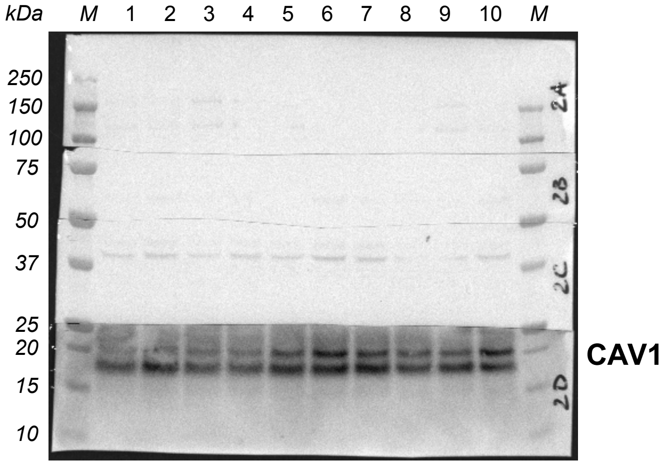

	αSMA	FSP1	FAPα	CAV1	CD90
**1**	24986.38	96974.56	24101.77	33276.17	56257.40
**2**	26089.10	79189.43	23552.81	31357.38	36932.62
**3**	25919.40	31549.58	22145.90	33647.50	43875.31
**4**	33036.94	8315.99	28072.27	31812.77	30663.22
**5**	25274.65	4932.87	33880.75	41278.65	36679.87
**6**	26870.46	6042.00	32460.24	54850.60	41749.12
**7**	35316.87	9990.07	27947.29	44721.82	40514.53
**8**	50461.65	8524.43	45481.82	38401.48	24637.97
**9**	28426.87	7209.82	21600.82	34601.41	58743.36
**10**	47403.95	7993.79	18309.65	43315.48	24689.41

lanes:
1: pat.5 NF cellular protein
2: pat.5 CAF cellular protein
3: pat.6 NF cellular protein
4: pat.6 CAF cellular protein
5: pat.7 NF cellular protein
6: pat.7 CAF cellular protein
7: pat.9 NF cellular protein
8: pat.9 CAF cellular protein
9: pat.10 NF cellular protein
10: pat.10 CAF cellular protein
M: Precision Plus Protein All Blue

Figure S5 A), part 3

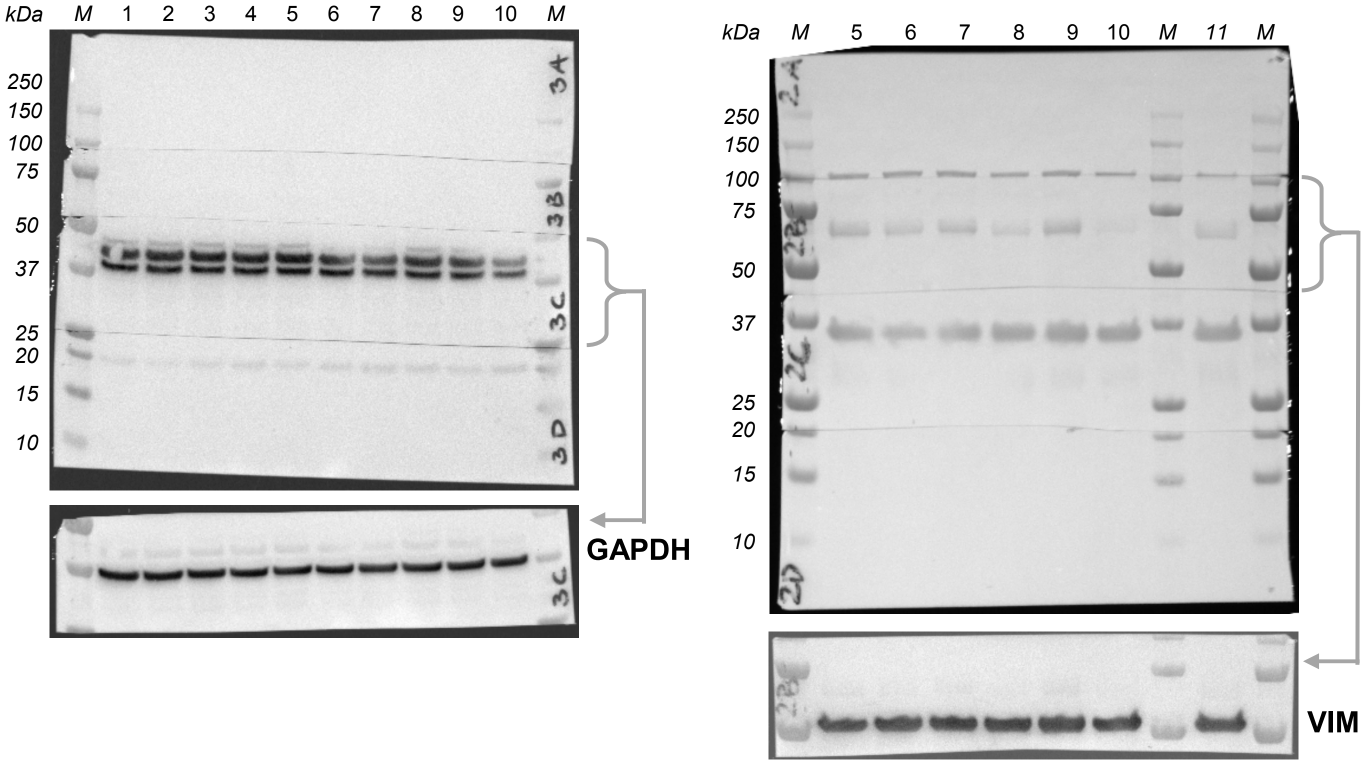

lanes:

1: pat.5 NF cellular protein
2: pat.5 CAF cellular protein
3: pat.6 NF cellular protein
4: pat.6 CAF cellular protein
5: pat.7 NF cellular protein
6: pat.7 CAF cellular protein
7: pat.9 NF cellular protein
8: pat.9 CAF cellular protein
9: pat.10 NF cellular protein
10: pat.10 CAF cellular protein
11: fibroblast cellular protein mix (2)
M: Precision Plus Protein All Blue

	VIM	GAPDH
**1**	-	88767.36
**2**	-	83131.58
**3**	-	77634.75
**4**	-	71036.17
**5**	50736.31	68480.87
**6**	53438.07	68618.75
**7**	56358.07	63402.29
**8**	57093.65	70563.82
**9**	60087.95	69855.46
**10**	55744.72	65747.41
**11**	60220.97	-

Figure S5 A), part 4

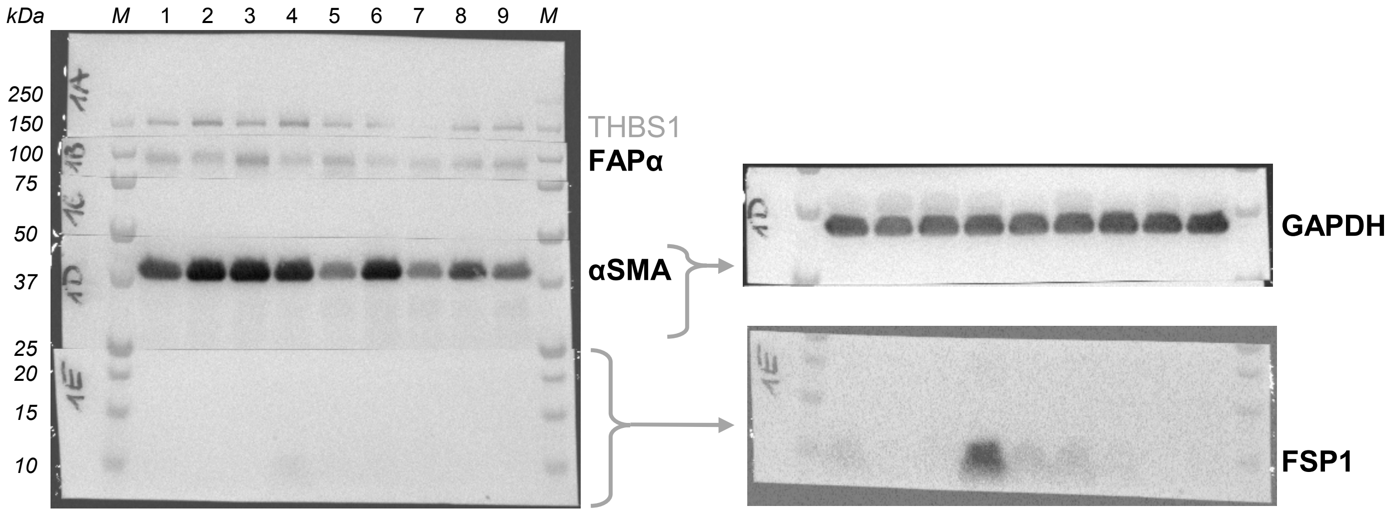

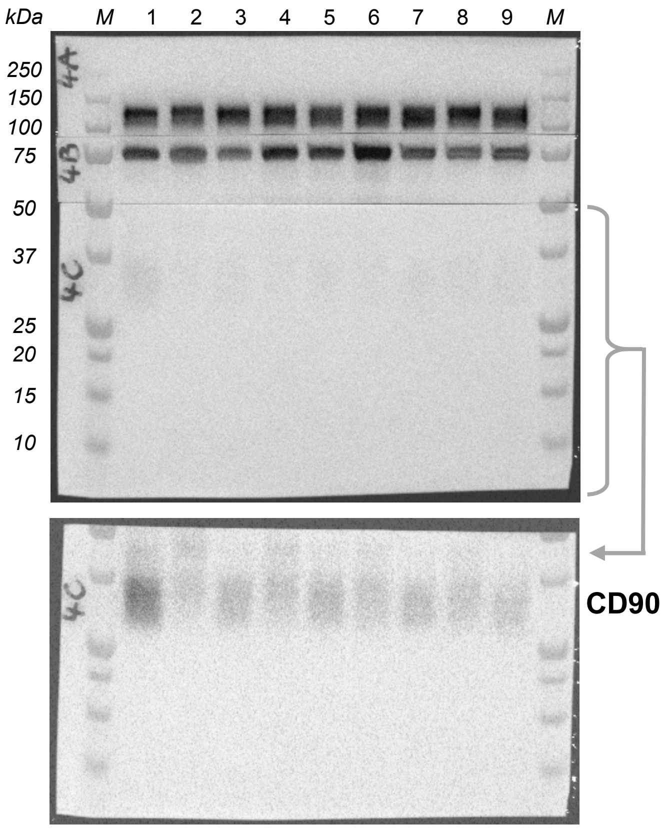

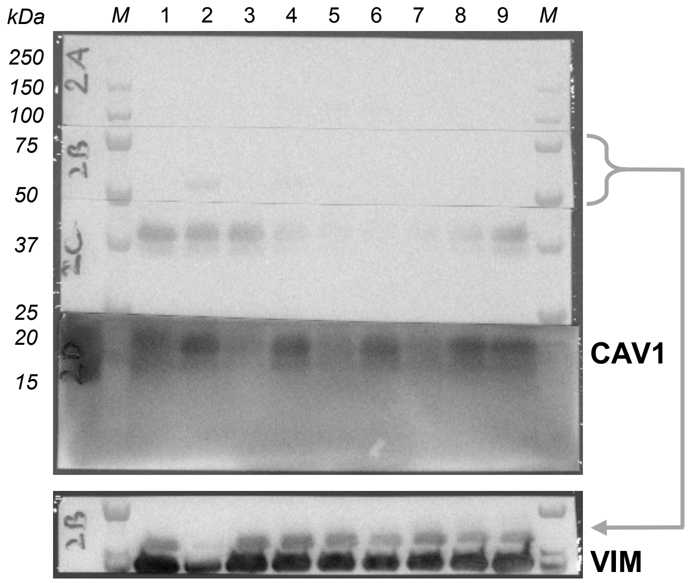

lanes:

1: pat.8 NF cellular protein
2: pat.8 CAF cellular protein
3: pat.11 NF cellular protein
4: pat.11 CAF cellular protein
5: pat.12 NF cellular protein
6: pat.12 CAF cellular protein
7: pat.13 NF_1 cellular protein
8: pat.13 CAF cellular protein
9: pat.13 NF_2 cellular protein

M: Precision Plus Protein All Blue

	αSMA	FSP1	FAPα	CAV1	VIM	CD90	GAPDH
1	63158.72	10064.36	52791.02	63935.17	87085.26	62502.72	62228.84
2	80621.36	2511.31	45654.48	64840.55	32170.87	13838.75	45707.82
3	83376.07	6203.65	74080.77	17306.95	76257.89	22574.07	54546.07
4	70696.77	72266.62	35800.82	41345.14	63403.95	17240.24	56949.65
5	29534.29	15683.75	48801.24	16195.41	56228.95	22879.65	53759.65
6	73536.72	15566.14	20257.12	38355.31	44623.82	15891.24	58157.36
7	25780.63	6947.53	17889.63	18212.29	53605.82	18947.77	65880.24
8	44655.53	3619.36	29718.82	48809.95	57998.70	15991.00	63999.12
9	32964.11	2429.41	42085.61	50764.77	71995.19	15493.84	76739.19
